# *Nandina domestica* Thunb.: a review of traditional uses, phytochemistry, pharmacology, and toxicology

**DOI:** 10.3389/fphar.2024.1407140

**Published:** 2024-07-09

**Authors:** Huiqin Qian, Yanling Li

**Affiliations:** College of Pharmacy, Sanquan College of Xinxiang Medical University, Xinxiang, China

**Keywords:** traditional uses, phytochemistry, pharmacology, toxicity, *Nandina domestica* Thunb.

## Abstract

Nandina domestica: Thunb. is a traditional Chinese herbal drug that has long been used in China and Japan for the treatment of colds, fevers, asthma, chronic bronchitis, conjunctivitis, whooping cough, pharyngeal tumors, etc. Published data have reported at least 366 constituents from *N. domestica*, including alkaloids, flavonoids, lignans, terpenoids, phenolic acids and their derivatives, fatty acids, and others. Of these, the isoquinoline alkaloids are considered characteristic markers for *N. domestica*. These alkaloids also showed the most promising bioactivities. The crude extracts or semi-purified constituents of *N. domestica* exhibit a variety of activities, including antitumor, dermatological, anti-inflammatory, antioxidant, antimicrobial, and detoxification activities, as well as effects on respiratory system, etc. The fruit is considered poisonous when eaten raw, with nausea, vomiting, diarrhea, and abdominal pain as side effects after ingestion. Most traditional uses are supported by biological activities demonstrated in modern experimental studies, suggesting a potential medicinal value of *N. domestica*. However, more information is needed on its mechanisms of activity, pharmacokinetic profile of the constituents, and its safety and efficacy profile in humans.

## 1 Introduction

**FIGURE 1:**
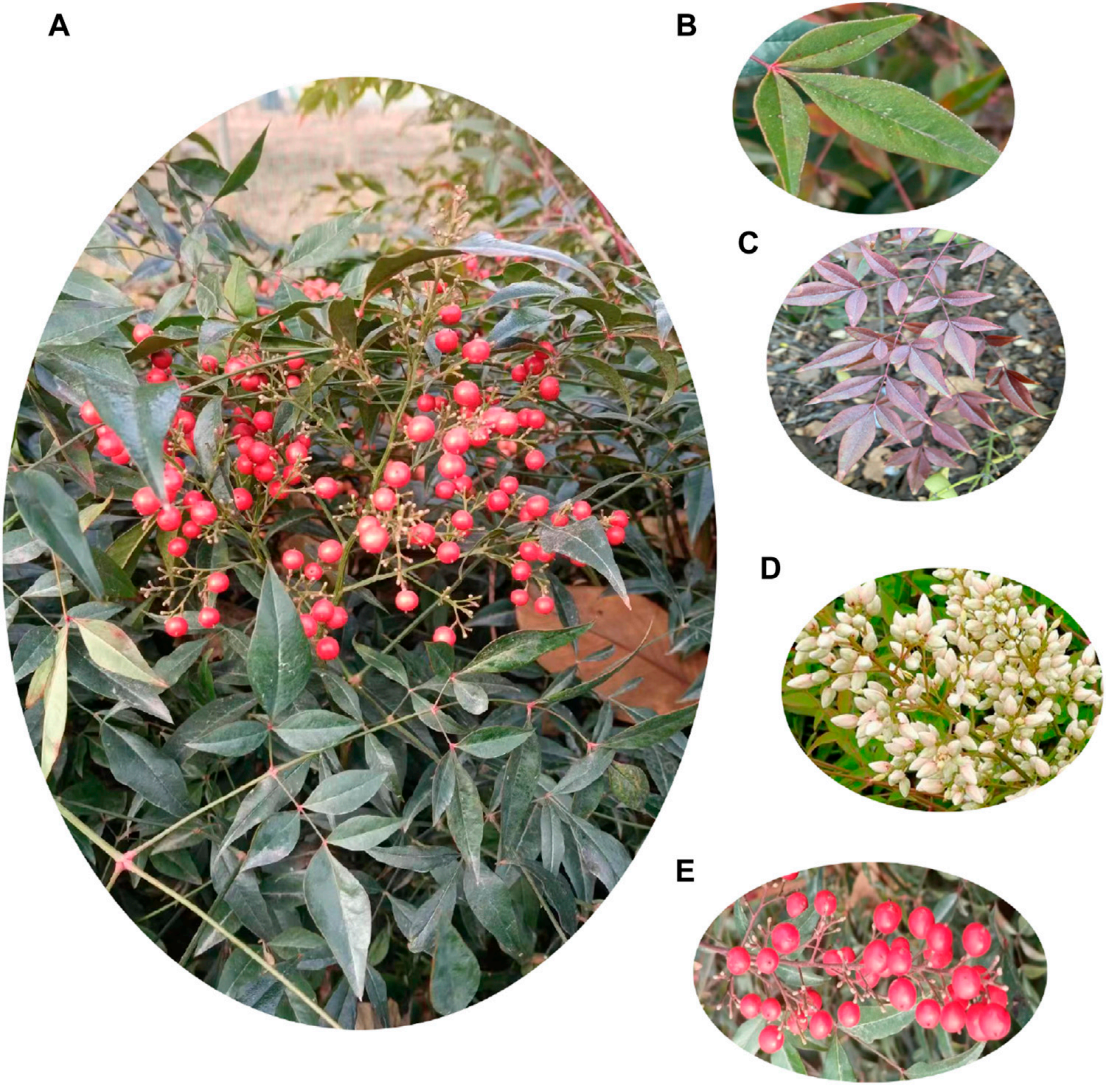
Plant images of *N*. *domestica*. **(A)** Whole plant. **(B)** Green leaves. **(C)** Red leaves. **(D)** Flowers. **(E)** Fruits.

Nandina domestica Thunb. belongs to the family Berberidaceae and is widely distributed in China, Japan, India, and Korea. *N. domestica* is frequently used in gardening as a landscape shrub. Its fruits ripen to a vivid red hue akin to coral and are grouped together like grapes. Its leaves have the appearance of bamboo leaves, and they can change from a beautiful green to a striking red in the fall and winter, giving the impression of a blazing fire (Tang, et al., 2007). The plant grows 2–3 m tall, with a typically cespitose stem that has few branches. Older branches are usually grey, whereas younger branches are often crimson. Leaves are habitually alternate, clustered in the upper part of the stem. Typically, leaves are commonly ternate-pinnate. The elliptic or elliptic-lanceolate leaflets have a form like feathers. The thin, leathery-textured leaflets have a rich green color that frequently becomes crimson in the winter. The tiny, white flowers have a strong, fragrant scent. Large panicles of flowers typically form at the apex of the stems and branches. The fruit is a berry and ripens in October-November. When the fruits reach maturity, they have a striking crimson color. Plant images of *N. domestica* are showed in Figure 1. Ecologically, *N*. *domestica* can adapt to limestone soil that is just slightly alkaline and significantly improve the ecological balance of the limestone regions that are otherwise not suitable to life. Besides, it also plays a vital role in greening the environment, purifying the air, conserving water, providing wind prevention and sand fixation, and maintaining soil and water (Liu, 2004). With regard to agricultural applications, *N*. *domestica* is an essential resource for the preparation of fungicides of botanical origin (Bajpai, et al., 2009b). Besides, *N. domestica*’s seeds are rich in fatty oils and can be used as raw material for extracting fatty oils (Liu, 2004).

*N. domestica* also possesses great medicinal values. In traditional Chinese medicine (TCM), its roots, stems, leaves, and fruits are used for clearing away heat and dampness, relieving cough and resolving phlegm, which is applied to treat symptoms such as cold and fever, whooping cough, asthma, chronic bronchitis, etc (Zhao, 1963; Xie, et al., 1983). The antifungal, antioxidant, anti–inflammatory, cytotoxic, and anti–platelet aggregation properties of *N. domestica*’s active ingredients have been verified by contemporary pharmacological investigations. Early findings indicate that the bioactive components of *N. domestic*a are isoquinoline alkaloids. In addition, *N. domestica* also contains lignans, flavonoids, triterpenoids, phenolic acids, and other compounds (Ishikura, 1971; Kunitomo, et al., 1972; Kodai, et al., 2010; Shu, et al., 2013; Yun, et al., 2021b; Qin, et al., 2021).

To date, there has been no comprehensive review on *N. domestica*. Therefore, this review thoroughly outlines the traditional applications, phytochemistry, pharmacological activity, and toxicity of *N. domestica* based on a large body of domestic and international literature, which provides guidance and reference for further research, utilization, and promotion of *N. domestica*.

## 2 Methodology

The existing scientific databases covering Google Scholar, Web of Science, PubMed, ScienceDirect, Elsevier, Wiley, Springer, Baidu Scholar, China Knowledge Resource Integrated Database (CNKI), Wanfang Data, and J-STAGE were used to gather the literature about the traditional uses, phytochemistry, and pharmacology of *N. domestica*. “*N. domestica*” in combination with “traditional uses,” “phytochemistry,” “pharmacology” was used as the search items from March 1949 to January 2024. ChemDraw 19.0 was used to draw the chemical structures.

## 3 Traditional uses

*N. domestica*, which is extensively distributed in China, Japan, India, and Korea, has been used as traditional herb for quite a long time. In China, the first record of *N. domestica* dates to the Song Dynasty’s “Tujing Ben Cao” (图经本草). This book recorded the plant morphology of *N. domestica* in detail and pointed out that it was often planted in the courtyard for ornamental purposes. The “Supplements to Compendium of Materia Medica” (本草纲目拾遗) from the Qing Dynasty recorded that *N. domestica* fruits could detoxify arsenic, as well as treating pubic lice and asthma. According to “Chinese Materia Medica” (中华本草), *N. domestica*’s roots, stems, branches, and leaves were bitter and cold in nature. According to its records, the roots are slightly toxic and have the function of clearing away heat and detoxification, removing dampness, expelling wind, resolving phlegm, and relieving cough. Its primary application is to treat lung-heat cough, damp-heat jaundice, diarrhea, rheumatic paralysis, sores, and scrofula. The stems, and branches function as a remedy for clearing dampness and heat and lowering rebellious qi, which are used to treat damp-heat jaundice, diarrhea, feverish gonorrhea, eye redness and swelling, cough and dysphagia. The leaves are known to be effective in clearing away heat and inducing dampness, diarrhea, and detoxification, which primarily treat lung-heat cough, whooping cough, febrile gonorrhea, blood in urine, swelling and pain in the eyes, sores and carbuncles, and scrofula. *N. domestica*’s fruits are sour and sweet in taste, and poisonous. Despite its toxicity, they are used to astringe lung for relieving cough, and reduce asthma. In Chinese medicine, they are used clinically to cure prolonged cough, wheezing and whooping cough. Moreover, in the “Jiangxi Province Chinese medicine concoction specification” (江西省中药炮制规范), the fruit of *N. domestica* was reported to have the effect of clearing the liver and brightening the eyes and was used for the treatment of malaria and skin ulcers. Furthermore, the roots, stems, leaves, and seeds of *N. domestica* are traditionally employed in China as folk medicine for the treatment of colds, fevers, asthma, chronic bronchitis, conjunctivitis, whooping cough, pharyngeal tumors, and uterine bleeding (Zhao, 1963; Xie, et al., 1983; Guo, et al., 2018). In Chinese medicine, *N. domestica* is frequently combined with other herbs for clinical applications. For instance, the combination of the stem and branches of *N. domestica* and the fibers of the mature fruits of *Luffa cylindrica* (L.) M. Roem. (family Cucurbitaceae), in addition to the pericarps of Lagenaria siceraria (Molina) Standl. (family Cucurbitaceae), is very effective in the treatment of acute and chronic nephritis (Dong, 1991). In addition, the fruits of *N. domestica*, the tuberous roots of Stemona japonica (Blume) Miq. (family Stemonaceae), and the pericarps of Cynanchum rostellatum (Turcz.) Liede & Khanum (family Apocynaceae) are combined for the treatment of acute and chronic or persistent prolonged cough, paroxysmal cough, coughing with no phlegm in severe coughing, and difficult coughing with little phlegm (Dai, 2007).

In Japan, the leaves, stems, and fruits of *N. domestica* are applied as folk medicine. The fruits, also called “nantenjitsu,” have been used to treat respiratory ailments such as asthma, whooping cough, and pharyngeal tumors (Takase and Ohashi, 1926). Additionally, the fruits are used to calm down inebriated people and treat impotence. Furthermore, the fruits are regarded as a therapeutic tonic that helped restore the neurological system’s equilibrium. A decoction of the leaves is ingested to treat fish and shrimp poisoning. The branches and leaves are reported to prolong life and the seeds strengthen manhood. Tuberculosis patients are treated with the roots’ aqueous extract (Lipp, et al., 1981). In addition, a throat lozenge called Nanten-nodo-ame, which contains extracts of the fruit of *N. domestica*, is being sold in the Japanese market (Okano, et al., 2017). In Korean folk medicine, the leaves are used to treat whooping cough, hematuria, and bruises. The fruits are effective in nourishing yin, clearing heat and tonifying qi, and are commonly used as a cough suppressant (Seo, et al., 2011).

## 4 Phytochemistry

Numerous in–depth phytochemical investigations have demonstrated that *N. domestica* generated a substantial number of secondary metabolites. Up to date, at least 366 compounds have been reported from the various parts of *N. domestica*, including alkaloids, flavonoids, lignans, terpenoids, phenolic acids and their derivatives, fatty acids, and others. *N. domestica*’s primary active components are isoquinoline alkaloids. Supplementary Table S1 displays the names, classifications, formulation, and other relevant information.

### 4.1 Alkaloids

**FIGURE 2:**
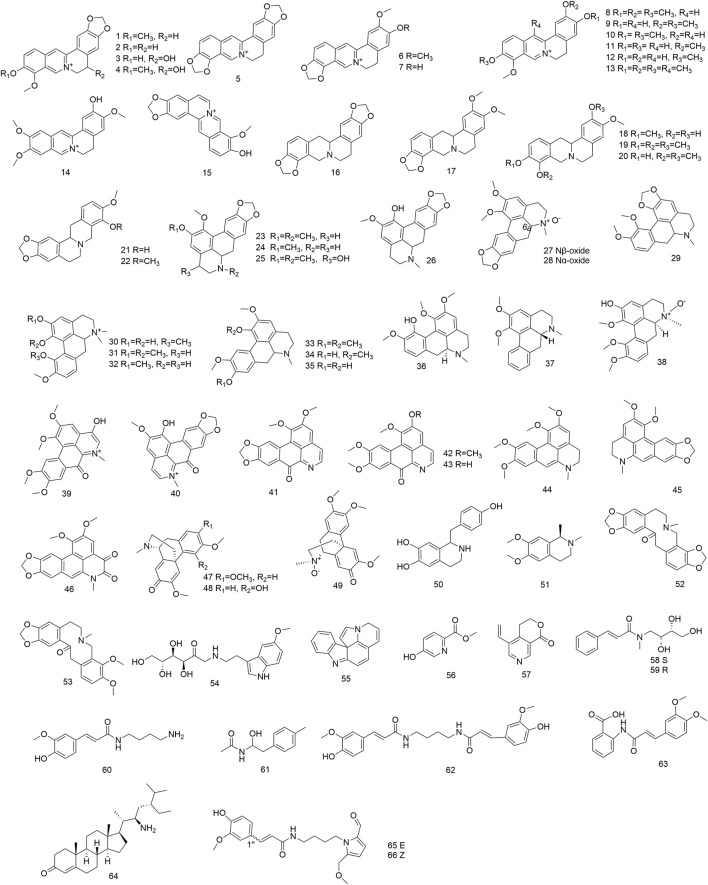
Structures of alkaloids (**1–66**) isolated from *N. domestica*.

Alkaloids are a class comprising many pharmacologically active constituents. Thus far, 66 alkaloids have been found in stems, fruits, and roots of *N. domestica.* Depending on their chemical structure, these alkaloids can be categorized as follows: isoquinoline alkaloids (**1–53**), indole alkaloids (**54–55**), pyridine alkaloids (**56–57**), organic amine alkaloids (**58–63**), steroidal alkaloids (**64**), and pyrrole alkaloids (**65–66**). Of these, isoquinoline alkaloids isolated from *N. domestica* are extensively studied. Their backbones are primarily made up of berberine (**1–15**), protoberberine (**16–22**), aporphine (**23–46**), morphinandienone (**47–49**), and tetrahydroisoquinoline (**50–51**). Aporphine–type alkaloids, like nantenine (**23**) and domesticine (**26**) have been documented to exhibit cytotoxicity against human tumor cells (Kunitomo, et al., 1974; Moriyasu, et al., 1992). A new aporphine–type alkaloid, isocorydine (**36**) was isolated from the fruits (Kunitomo, et al., 1972). Peng et al. successively isolated a new steroidal alkaloid, nandsterine (**64**) coupled with two novel organic amine alkaloids, *N*–methyl–3–phenyl–*N*–[2(*S*),3*R*,4–trihydroxy–butyl]–acrylamide (**58**) and *N*–methyl–3–phenyl–*N*–[2(*R*),3*R*,4–trihydroxy–butyl]–acrylamide (**59**) (Peng, et al., 2014b; Peng, et al., 2020). Furthermore, two novel aporphine–type isoquinoline alkaloids, 6*R*,6a*S*–*N*–nantenine *N*_*β*_–oxide (**27**) and 6S,6aS–*N*–nantenine *N*_*α*_–oxide (**28**), were extracted from the seeds (Qin, et al., 2021). A phytochemical investigation of methanol extract of the fruits furnished two new pyrrole alkaloids, namely methyl–*E*–mangolamide (**65**) and methyl–*Z*–mangolamide (**66**). These compounds exhibited cell death–inducing activity against adriamycin–treated HeLa cells (Imahori, et al., 2021). According to the aforementioned–research, *N. domestica* is a natural resource for the extraction of alkaloids. Nevertheless, it is presently uncertain which alkaloids could be utilized as indicators of medicinal quality. Figure 2 presented the structures of the alkaloids (**1–66**) that were isolated from *N. domestica*.

### 4.2 Flavonoids

**FIGURE 3:**
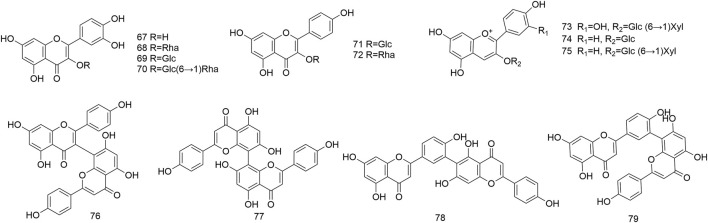
Structures of flavonoids (**67**–**79**) isolated from *N. domestica*.

Thirteen flavonoids (**67–79**) were discovered primarily from the leaves and fruits of *N. domestica* (Figure 3). These compounds are separated into flavonols (**67–72**), anthocyanins (**73–75**) as well as biflavones (**76–79**).

According to earlier studies, fruits were discovered to contain anthocyanins (**73–75**), including cyanidin 3–xylosylglucoside (**73**), pelargonidin 3–glucoside (**74**), and pelargonidin 3–xylosylglucoside (**75**) (Ishikura, 1971). Under the guidance of the anti-inflammatory activity, robustaflavone (**78**), a biflavone, was extracted from the methanol extract of *N. domestica* fruits (Jo, et al., 2019). Further, amentoflavone (**79**) was isolated from the leaves and fruits, which demonstrated notable antioxidant property and was able to inhibit the growth of *Staphylococcus aureus* and *Escherichia coli* (Bajpai, 2019). Currently, there is no research on the types and contents of flavonoids in the various medicinal parts of *N. domestica*. Thus, additional studies are essential to thoroughly investigate the flavonoids of *N. domestica*.

### 4.3 Lignans

**FIGURE 4:**
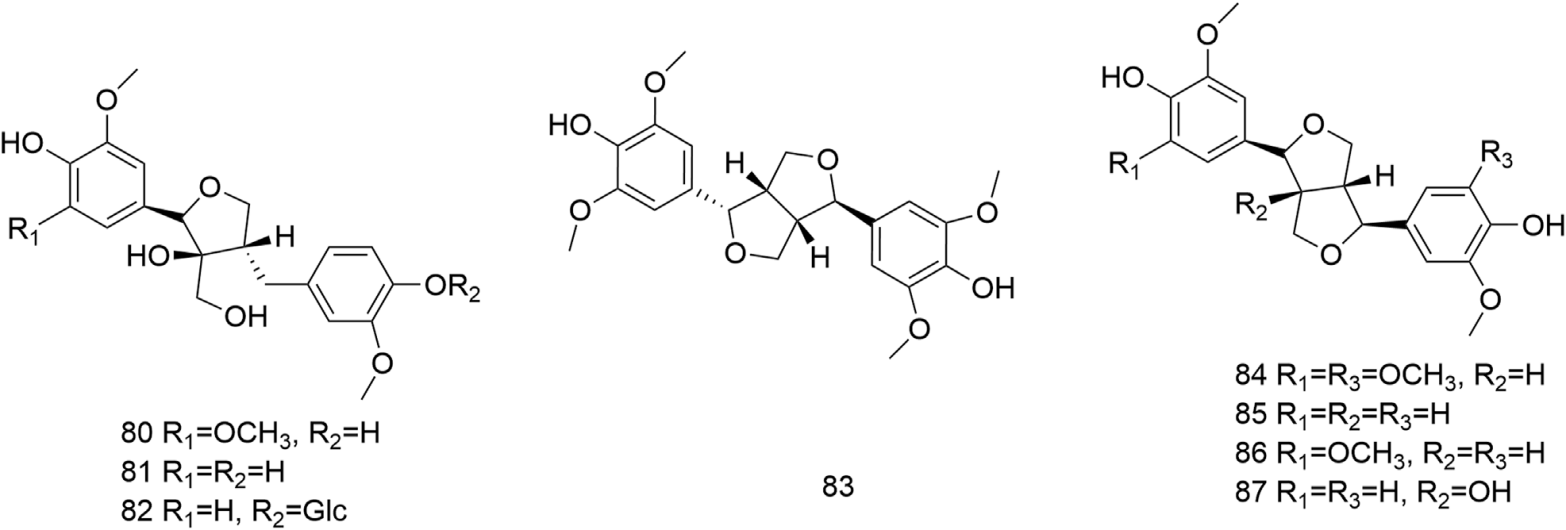
Structures of lignans (**80**–**87**) isolated from *N. domestica*.

Only eight lignans (**80–87**) have been isolated and identified from *N. domestica*, comprising tetrahydrofurans (**80–82**) and furofurans (**83–87**) (Figure 4). Hence, the structural variety of lignans is restricted. Notably, (−)–episyringaresinol (**83**) was isolated from tree bark (Kunitomo, et al., 1975). Seven lignans were separated from 80% ethanol extract of the seeds of *N. domestica*, including gentioluteol (**80**), berchemol (**81**), berchemol–4′–*O*–*β*–D–glucoside (**82**), syringaresinol (**84**), pinoresinol (**85**), medioresinol (**86**), along with 1–hydroxypinoresinol (**87**). The physicochemical characteristics, NMR spectroscopy data analysis, along with comparisons with published reports were utilized to clarify their structures (Shu, et al., 2013).

### 4.4 Terpenoids

**FIGURE 5:**
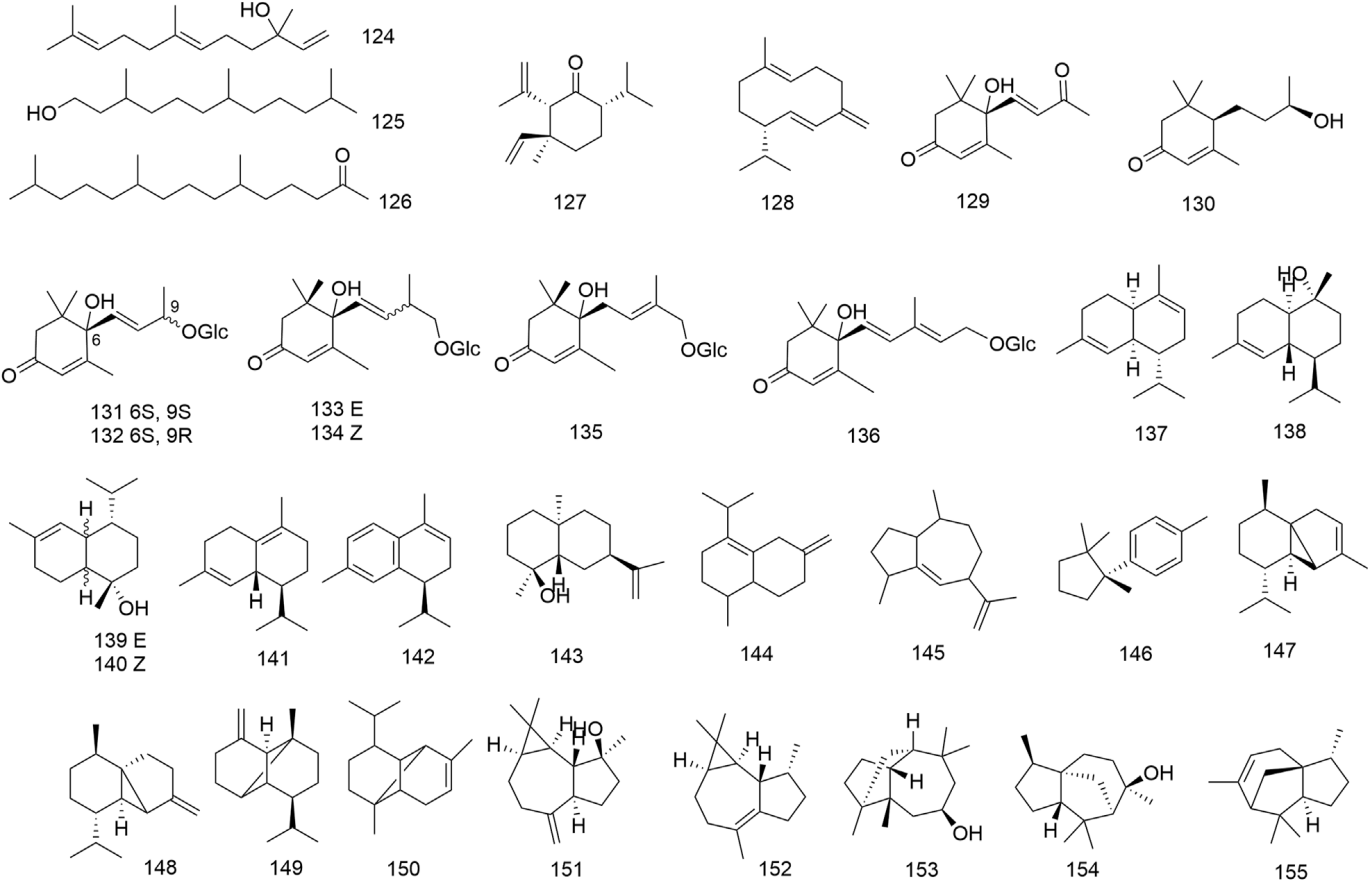
Structures of sesquiterpenoids (**124**–**155**) isolated from *N. domestica*.

A total of seventy–one terpenoids (**88–158**) have been primarily discovered from methanol extract of *N. domestica* leaves and fruits, which cover monoterpenoids (**88–123**), sesquiterpenoids (**124–155**), diterpenoids (**156–157**), as well as triterpenoids (**158**). Notably, all the monoterpene compounds (**89–116**, **118–123**) apart from loliolide (**117**) were reported from the volatile oil of *N. domestica* by gas chromatography–mass spectrometry (GC–MS). One of the predominant components of *N. domestica*’s volatile oil was linalool (**88**) (Bi, et al., 2016). Bajpai et al. discovered that the volatile oil of flowers consists of mainly oxygenated mono- and sesquiterpenes, and mono- and sesquiterpene hydrocarbons. Additionally, the volatile oil of flowers was able to control food–borne pathogenic bacteria, and agricultural plant diseases (Bajpai, et al., 2008; Bajpai, et al., 2009b). In general, sesquiterpenoids can be distinguished on the backbone differences in the biosynthesis. The predominant skeletal type of sesquiterpenes isolated from *N. domestica* is megastigmane–type (**129–136**) and eudesmane–type (**137–144**). Nandinamegastigmanes I–IV (**133–136**), four new megastigmane glycosides, were extracted from the methanol extract of the fruits (Imahori, et al., 2021). An undescribed cycloartane–type triterpenoid, called 24–methylene–3–oxocycloartane 13–carboxylic acid (**158**) was purified from a methanol extract from the fruits (Kodai, et al., 2010). The structures of sesquiterpenoids (**124–155**) are displayed in Figure 5.

### 4.5 Phenolic acids and their derivatives

**FIGURE 6:**
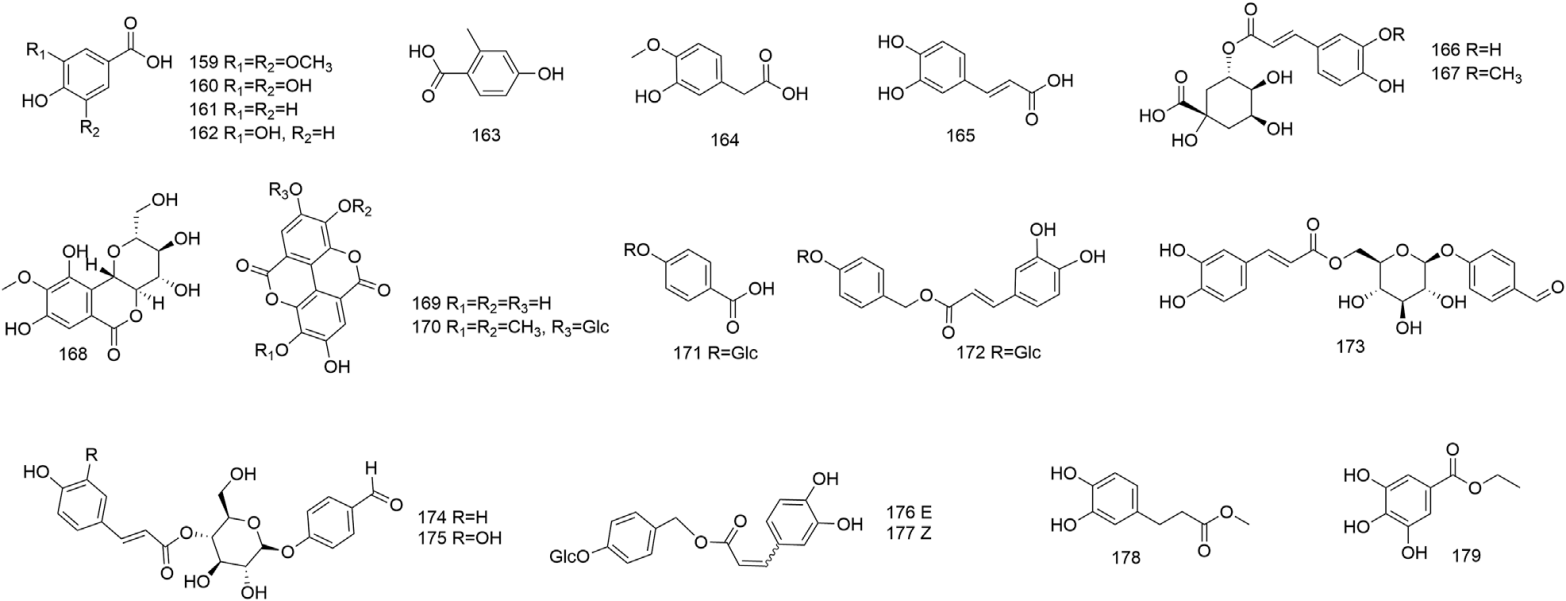
The structures of phenolic acids and their derivatives (**159–179**).

Twenty–one phenolic acids and their derivatives were reported from roots, leaves, flowers, fruits, and seeds of *N. domestica*, comprising phenolic acids (**159–169**), phenolic glycosides (**170–177**), and phenolic acid esters (**178–179**). Nine phenolic compounds were obtained from the ethanol extract of the seeds and their structures were elucidated as syringic acid (**159**), gallic acid (**160**), *p*–hydroxybenzoic acid (**161**), protocatechuic acid (**162**), caffeic acid (**165**), bergenin (**168**), ellagic acid (**169**), 3,3′–di–*O*–methylellagic acid–4–*O*–*β*–D–glucoside (**170**), as well as ethyl gallate (**179**) (Peng, et al., 2014c). Initially, two phenolic acids, caffeic acid (**165**) and chlorogenic acid (**166**), were extracted and identified from 70% ethanol extract of leaves (Taha, et al., 2019). Under the guidance of tyrosinase inhibition activity, Masuda et al. extracted a simple phenolic glucoside, 4–*β*–D–glucopyranosyloxybenzoic acid (**171**), from the ethanol extract of the leaves (Masuda, et al., 2007). Moreover, Kulkarni et al. et al. have reported the isolation and characterization of one new caffeoyl glucoside, nandinaside A (**173**), as well as two known caffeoylated compounds, nantenoside B (**175**), gastrodin–7–*O*–*trans*–caffeoyl ester (**172**), from the methanol extract of fruits (Kulkarni, et al., 2015). Two known phenol glucosides, 4–*O*–*β*–D–glucopyranosylbenzyl–(*E*)–3–(3,4–dihydroxyphenyl)acrylate (**176**), and 4–*O*–*β*–D–glucopyranosylbenzyl–(*Z*)–3–(3,4–dihydroxyphen–yl)acrylate (**177**), were identified from the methanol extract of the fruits (Imahori, et al., 2021). Figure 6 depicts the structures of phenolic acids and their derivatives.

### 4.6 Fatty acids

**FIGURE 7:**
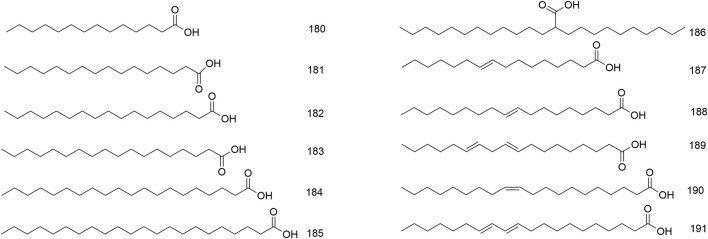
Structures of fatty acids (**180**–**191**).

**FIGURE 8:**
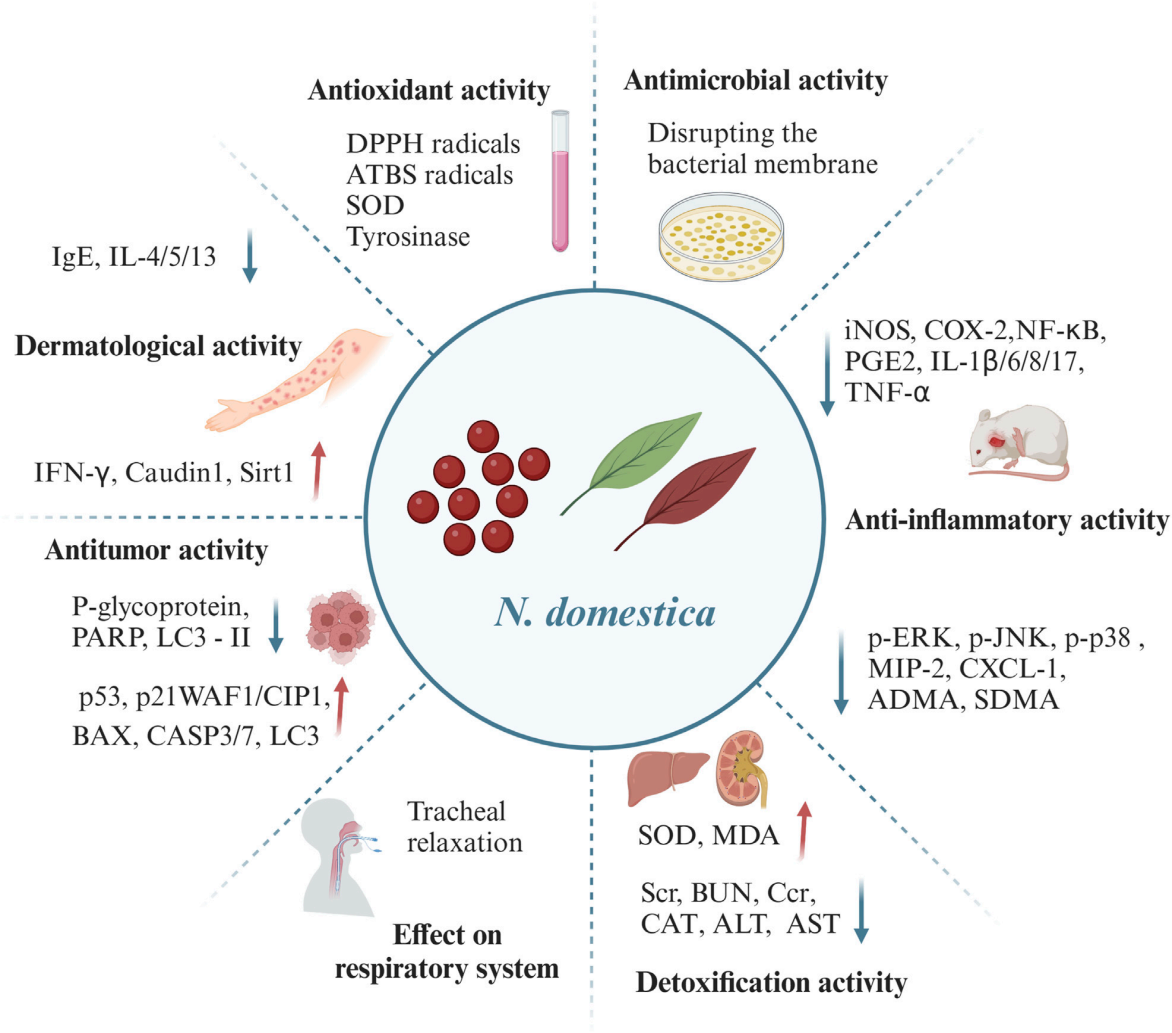
Pharmacological activities of *N. domestica* (Red arrows denote upward adjustments, and blue arrows denote downward adjustments).

Fatty acids were principally present in the seeds of *N. domestica*. Moreover, its leaves, flowers and fruits also contained fatty acids in their volatile oil (Wang, et al., 2022; Zhang, et al., 2014). To date, twelve fatty acids, covering saturated (**180–186**) and unsaturated (**187–191**) fatty acids, were detected from *N. domestica* (Figure 8). One study pointed out that the oil content of the seeds was12.26%. A total of 13 fatty acids were determined by GC–MS. Of them, unsaturated fatty acids were the predominant components of the seed oils, which were mainly composed of palmitic acid (**181**), stearic acid (**183**), oleic acid (**188**) and linoleic acid (**189**) (Wang, et al., 2014). The fatty acids (**180–191**) are shown in Figure 7.

### 4.7 Others

In addition to the mentioned-above chemicals, various medicinal parts of *N. domestica* have been shown to contain hydrocarbons (**191–230**), alcohols (**231–264**), ketones (**265–277**), carboxylic acids (**278–283**), ethers (**284–295**), aldehydes (**296–313**), esters (**314–338**), *N*–containing compounds (**339–363**), and other compounds (**364–366**). Notably, the majority of the above-mentioned compounds were detected from the volatile oils of *N. domestica* using gas chromatography–mass spectrometry (GC–MS) (Bi, et al., 2016). Young shoots have been reported to furnish a novel cyanogenic glucoside, *p*–glucosyloxy–mandelonitrile (**362**) (Abrol, et al., 1966). Additionally, a new cyanogenic compound, nandinin (**363**), was obtained from the methanol extract of young leaves (Olechno, et al., 1984). A growth inhibitory substance known as *p*–hydroxybenzaldehyde (**311**) was isolated from the methanol extracts (Han, et al., 2011). Besides, the enzymes, protein, amino acids, and trace elements were also obtained from *N. domestica.* Two hydroxynitrile lyases with molecular mass of 24.9 kDa (NdHNL–S) and 28.0 kDa (NdHNL–L) were isolated from the young leaves containing a new amino acid sequence and possessing the potential for efficient production of (*R*)–cyanohydrins (Isobe, et al., 2018). Furthermore, superoxide dismutase and peroxide isoenzymes were also present in the fresh leaves of *N. domestica* (Tang, et al., 2008). The leaves had amino acids like Lys, Met, Phe, Val, Leu, Ile, Thr, His, Arg, Asp, and others, as well as proteins like albumin, globulin, gliadin, and glutenin (Niu, et al., 2013). Ten trace elements, including Mo, Co, Cr, Ni, Cd, Pb, Fe, Cu, Zn, and Mn, were found in the roots, stems, and leaves of *N. domestica*. Among them, the content of Pb and Cd was low. In contrast, the content of the eight remaining trace elements was high (Shu, et al., 1988).

## 5 Pharmacology

*N. domestica* has long been used therapeutically in China and Japan for treating lung-heat cough, whooping cough, wheezing, asthma, chronic bronchitis, conjunctivitis, sores, and scrofula, etc. Pharmacological experiments have disclosed that *N. domestica* extracts, volatile oils and its isolated compounds exhibited significant pharmacological activities, such as effect on respiratory system, anti–inflammatory, dermatological, and detoxification activities, as discovered in traditional uses. Apart from this, its other significant pharmacological activities have been reported, including antitumor, antioxidant, as well as, antimicrobial activities, etc. Supplementary Table S2 and Figure 8 present the specific pharmacological activity.

### 5.1 Antitumor activity

The incidence of malignant tumors is rising annually around the world owing to several factors, including chronic infections, dietary habits, environmental pollution, and increasing in the age of the global population. Malignant tumors currently remain a major global public health issue. Current medications for the treatment of malignant tumors have several drawbacks, including poor efficacy, adverse effects, drug resistance, and high costs. The treatment of malignant tumors requires the use of safe, efficient pharmaceuticals or molecules derived from natural remedies. The roots, stems, leaves, and seeds of *N. domestica* are traditionally employed to treat pharyngeal tumors. An *in vivo* study demonstrated that chloroform and methanol extracts of rootbarks exhibited comparatively strong *in vitro* cytotoxic activity against P388 leukaemia cells with IC_50_ of 1.5 and 3.0 μg/mL, respectively. Subsequently, berberine (**1**) was extracted from the aforementioned extracts and demonstrated a noteworthy cytotoxic activity against P388 leukaemia cells with IC_50_ of 0.4 μg/mL (Funayama, et al., 1996).

The present studies on the antitumor activity of *N. domestica* are concentrated on its alkaloids and only for *in vitro* studies. A new alkaloid, nandsterine (**64**) isolated from the fruits, demonstrated cytotoxicity against human leukemia HL–60 cells with IC_50_ values of 52.1 μM (Peng, et al., 2014b). Similarly, oxonantenine (**41**) was remarkably cytotoxic to A549 cells with an IC_50_ value of 8.15 ± 0.34 μM. Nantenine (**23**) and nornantenine (**24**) were moderately cytotoxic to A549 cells with IC_50_ values of 58.94 ± 2.81 and 48.98 ± 2.57 μM, respectively (Qin, et al., 2021). Two new pyrrole alkaloids, methyl–*E*–mangolamide (**65**) and methyl–*Z*–mangolamide (**66**) demonstrated cell–induced death in adriamycin–treated HeLa cells. Mechanically, P–glycoprotein inhibition might be associated with the process of inducing cell death (Imahori, et al., 2021). Protopine (**52**), an isoquinoline alkaloid, prevented the growth of colon cancer cells by stabilizing p53, which in turn caused apoptosis and autophagy in colon cancer cells. Specifically, protopine (**52**) increased p53–mediated transcriptional activity in a dose–dependent manner, improved the phosphorylation of p53 at position Ser15, which helped to stabilize the p53 protein. Moreover, protopine (**52**) upregulated the expression of p53 downstream genes p21WAF1/CIP1 and BAX. Protopine (**52**) suppressed dose–dependently the proliferation of colon cancer HCT116 cells, activated caspase 3/7 (CASP3/7), which subsequently catalyzed poly ADP–ribose polymerase (PARP) cleavage and increased the number of annexin V–FITC–positive cells. Besides, protopine (**52**) stimulated autophagy in tumor cells via inducing microtubule–associated protein 1 light chain 3 (LC3) puncta generation and LC3–II conversion (Son, et al., 2019). Taken together, the alkaloids of *N. domestica* showed anti–tumor activity.

### 5.2 Dermatological activity

Atopic dermatitis (AD), occasionally referred to as atopic eczema, is a recurring, itchy, and chronic inflammatory skin disease. In China, *N. domestica* has been used for the treatment of sores and scrofula. From the perspective of Western medicine, sores and scrofula are understood to be purulent skin diseases. From the perspective of Chinese medicine treatment, heat-clearing and detoxification drugs are frequently used to treat sores and scrofula. Notably, *N. domestica*’s leaves have the effect of clearing heat and removing toxins, which might be used for sores and scrofula. Their application has also been proven in several modern research studies. In a 1–chloro–2,4–dinitrobenzene (DNCB)-induced atopic dermatitis model in NC/Nga mice, 70% ethanol extract of leaves treatment at the dose of 300 mg/kg for 5 weeks normalized the total cell number (TCN) in the spleen, axillary lymph node (ALN), and dorsal skin tissue while lowering blood immunoglobulin E (IgE) levels. Furthermore, the experiments revealed a noteworthy reduction in the quantity of CD23+/B220+ cells in the ALN and CD3^+^ cells in the dorsal skin, as well as a decline in the levels of interleukin (IL)–4, IL–5, and IL–13. Besides, the splenocytes’ expression of interferon–*γ* (IFN–*γ*) was elevated, and the claudin1 and sirt1 protein expression was notably upregulated. Taken together, 70% ethanol extract of leaves remarkably improved DCNB–induced AD (Yun, et al., 2021b). A similar study found the ethyl acetate fraction of the 70% ethanol extract from the leaves exhibited anti–eczematic activity against DNCB induced eczema in mice, which was superior to the positive control mometasone furoate in the treatment of eczema (Taha, et al., 2019).

The antidermatophytic activity of the essential oil and several chemical extracts (*n*–hexane, chloroform, ethyl acetate, and methanol) from flowers was assessed against fungal pathogens that caused skin infections, such as *Trichophyton rubrum*, *Trichophyton mentagrophytes*, *Microsporum canis*. The essential oils demonstrated moderate to high antidermatophytic efficacy against all tested dermatophytes with inhibition of 51.90%–68.60%. Furthermore, essential oils displayed a strong suppression of spore germination in all tested dermatophytes and revealed a concentration–and time–dependent kinetic inhibition of *Trichophyton rubrum*. Additionally, the extracts showed mild to moderate antidermatophytic effect against fungal pathogens that cause skin infections with inhibition of 19.2%–55.1%. Taken together, essential oils and extracts could serve as a natural fungicide to treat skin fungus infections (Bajpai, et al., 2009a). To summarize, the different extracts of *N. domestica* produced promising anti–eczema and anti–skin fungal activities. Nevertheless, further research is needed to determine the main active ingredients in the extracts of *N. domestica*.

### 5.3 Anti–inflammatory activity

Inflammation is a defense response of the immune system to infection or injury. It can get rid of dangerous substances and damaged tissue but can also lead to tissue damage and malfunction. *N. domestica* has been used for many years in the treatment of inflammatory respiratory diseases such as asthma, whooping cough, and pharyngeal tumors. Existing modern investigations employing lipopolysaccharide (LPS)–induced models of inflammation have validated the anti–inflammatory properties of the extract of *N. domestica* and its active ingredients. A study demonstrated that aqueous extract from *N. domestica* fruits treatment at concentrations of 1∼10 μg/mL suppressed dose–dependently the expression of cyclooxygenase–2 (COX–2) and the production of prostaglandin E2 (PGE2) in LPS–stimulated human pulmonary epithelial A549 cells without affecting COX–1 expression and COX activity (Ueki, et al., 2012). A recent study investigated that 70% ethanol extract of *N. domestica* leaves decreased nitric oxide (NO) production and inhibited interleukin (IL)–6 and IL–1*β* mRNA expression in LPS–stimulated RAW 264.7 macrophages in a dose–dependent manner. 70% Ethanol extract suppressed phosphorylated activation of mitogen–activated protein kinase (MAPK) signaling pathways, encompassing extracellular signal–regulated kinase (ERK), p38 and c–Jun N–terminal kinase (JNK). Mechanistic studies indicated that 70% ethanol extract exerted anti–inflammatory effects by mediating MAPK phosphorylation and down–regulating inflammatory mediator levels (Yun, et al., 2020). An investigation using a fine dust mixture of coal, flay ash, and diesel exhausted particle (CFD)–induced lung injury mice model demonstrated that 70% ethanol extract of leaves reduced lung injury through suppressing inflammatory cytokines and reducing neutrophil accumulation. To be more precise, 70% ethanol extract of leaves suppressed the level of inflammatory cytokines such as IL–17, tumor necrosis factor–alpha (TNF–α), macrophage inflammatory protein–2 (MIP–2), and C–X–C motif chemokine 1 (CXCL–1) and dramatically decreased neutrophil counts. Furthermore, the 70% ethanol extract led to improvements in lung tissue, such as goblet cell hyperplasia and inflammatory cell infiltration, based on a histological assessment. Also, the elevation of symmetric dimethylarginine (SDMA) and asymmetric dimethylarginine (ADMA) levels was suppressed by 70% ethanol extract (Yun, et al., 2021a). In LPS–stimulated RAW 264.7 macrophages, the pro–inflammatory cytokines IL–1*β*, IL–6, and NO were reduced by the administration with robustaflavone (**78**). Furthermore, robustaflavone (**78**) suppressed the expression of inducible nitric oxide synthase (iNOS) and COX–2, and down–regulated nuclear factor–κB (NF–κB) expression and phosphorylation of extracellularly regulated kinases (pERK 1/2). All the findings demonstrated that robustaflavone (**78**) had anti–inflammatory property (Jo, et al., 2019). Besides, in LPS–mediated endothelial inflammatory responses elicited in human umbilical vein endothelial (HUVEC) cells, two caffeoyl glucosides, nandinaside A (**173**), and nantenoside B (**175**), dose–dependently suppressed LPS–induced leukocyte hyperpermeability, adhesion and migration on human endothelial cell monolayers (Kulkarni, et al., 2015). From the studies mentioned above and their remarkable results, extracts from *N. domestica* and its active compounds has anti–inflammatory properties. It is still necessary to thoroughly investigate additional anti–inflammatory components and conduct extensive animal model studies to fully understand the anti–inflammatory effect of *N. domestica* extract.

### 5.4 Antioxidant activity

The extracts of *N. domestica* are confirmed to exhibit certain antioxidant activity by various antioxidant methods such as DPPH, ABTS, and superoxide radical scavenging, reducing power, and metal chelating ability assays. Some studies have shown that 70% ethanol extract and 80% methanol extract of *N. domestica*’s leaves dose–dependently possessed favourable DPPH radical scavenging activity (He, et al., 2003; Yun, et al., 2020). Moreover, ethanol extract of leaves demonstrated exceptionally high tyrosinase inhibitory action at 0.5 and 0.15 mg/mL, with inhibitory rates of 81.5% ± 2.0% and 65.7% ± 1.7%, respectively (Masuda, et al., 2007). One study demonstrated that the volatile oil from the flowers possessed good scavenging effects on ABTS radicals as well as metal chelating ability in a concentration–dependent manner (Zhang, et al., 2014). Another similarity study confirmed that the volatile oil from fruits exhibited relatively high ferric ion reducing power with an IC_50_ value of 145.35 ± 4.10 μg/mL, which was equivalent to that of ascorbic acid. Moreover, the volatile oil of the fruits displayed pronounced scavenging activity against DPPH and ATBS radicals with IC_50_ values of 28.39 ± 1.12 and 20.61 ± 0.75 μg/mL, respectively. The oil showed moderate scavenging activity against superoxide radicals with an IC_50_ value of 53.22 ± 2.51 μg/mL. However, its anti-metal chelating activity was low with an IC_50_ value of 92.5 ± 3.16 μg/mL (Bi, et al., 2016). Besides, amentoflavone (**79**) displayed potent antioxidant activity on scavenging DPPH, ABTS, superoxide, and hydroxyl radicals in a concentration–dependent manner with inhibition ranging from 19.21% to 75.52% (Bajpai, et al., 2019). According to the aforementioned findings, *N. domestica* exhibits favourable antioxidant activity and may be employed as a functional material with antioxidant properties.

### 5.5 Antimicrobial activity

The extracts of *N. domestica* stems have been found to have antibacterial effects on *S. aureus*, *Streptococcus faecalis*, *B. thuringiensis*. Meanwhile, the extracts of *N. domestica* leaves displayed antibacterial activity against *Bacillus thuringiensis* (Li, 2007; Li, et al., 2008). Moreover, one study showed that aqueous extract of leaves reduced the growth of both Gram-positive (*S. aureus*, *S. pyogenes*) and Gram-negative bacteria (*E. coli*, *P. aeruginosa*, *A. baumannii*) by disrupting the bacterial membrane. The aqueous extracts of the leaves were further separated into ethanol, petroleum ether, and ethyl acetate fractions, in which the ethyl acetate fraction exhibited the highist antibacterial activity. Using antimicrobial activity–guided fractionation, alkaloids and flavonoids were isolated from leaf extracts. Antimicrobial tests revealed that the alkaloids inhibited Gram-positive bacteria, whereas the flavonoids suppressed Gram-negative bacteria (Guo, et al., 2018). The aforementioned tests demonstrate the antibacterial activity of *N. domestica*’s varous extract. Additional studies may be conducted to determine the active components of the various extracts and their safety as medications.

### 5.6 Detoxification activity

Arsenic trioxide is used internationally as one of the targeted drugs for the treatment of acute promyelocytic leukaemia. However, arsenic trioxide inhibits antioxidant enzyme activities such as glutathione or selectively enhanced cytochrome P450–dependent monooxygenase activity during therapy, which results in oxidative damage to the liver and kidneys, which severely limits its use in clinical practice. Therefore, there is a critical need to discover the specific components responsible for the detoxifying effect. In the Qing Dynasty’s “Supplements to Compendim of Materia Medica” (本草纲目拾遗), it was documented that the aqueous extract of *N. domestica’s* fruits could detoxify arsenic. Many modern studies have been conducted to evaluate the detoxification effects of *N. domestica* on the antitumor drug arsenic trioxide using a chronic arsenic trioxide-induced hepatotoxicity and nephrotoxicity in rats. The findings showed that aqueous extract of seeds decreased arsenic trioxide-induced hepatotoxicity and nephrotoxicity. Concretely, aqueous extract of seeds treatment at the dose of 20 g/kg reduced structural damage to liver and renal tissue without hepatocellular necrosis, lowered alanine aminotransferase (ALT), aspartate aminotransferase (AST), serum creatinine (Scr), and blood urea nitrogen (BUN) levels, attenuated the decrease in endogenous creatinine clearance rate (Ccr), decreased catalase (CAT) levels and increased superoxide dismutase (SOD) and malondialdehyde (MDA) levels in the homogenates of the renal cortex, which compared to the untreated group (Peng, et al., 2014a; Liu, et al., 2015). Interestingly, 70% ethanol extracts of roots, stems, and fruits showed significant protective effects against arsenic trioxide oxidative stress-induced hepatotoxicity and nephrotoxicity, whereas *N. domestica*’s leaves were less effective (Sun, et al., 2019). A similar study demonstrated that total alkaloids from a 70% ethanol extract significantly ameliorated arsenic trioxide-induced cardiac, renal, and hepatic damage. Accordingly, total alkaloids were considered to present an attenuating effect on arsenic trioxide toxicity. Notably, the aporphine alkaloids, such as nantenine (**23**) and domesticine (**26**), were characterized by comparatively high content and high specificity, which could serve as indicator components for the quality control methods of *N. domestica* with regards to the cardiac, renal, and hepatic protective affords (Cheng, et al., 2020). Besides, a recent study demonstrated that different fractions (chloroform, ethyl acetate, *n*–butanol and aqueous) from the 70% ethanol extract of roots exhibited a favourable protective effect against arsenic trioxide–induced hepatotoxicity and nephrotoxicity. Notably, the most pronounced antagonistic effect on toxicity was observed with the chloroform fraction. Meanwhile, berberine (**1**), the principal component of the chloroform fraction, showed a dose–dependent protective effect against arsenic trioxide–induced hepatotoxicity and nephrotoxicity (Fu, et al., 2023). Based on the above–mentioned results, the extracts from *N. domestica* exhibit detoxifying effects on arsenic trioxide induced nephrotoxicity and hepatotoxicity.

### 5.7 Effect on respiratory system

Asthma is characterized by severe airway constriction brought on by airway inflammation. For the treatment of asthma, glucocorticosteroids are currently the first option. However, a safer and more effective asthma medication, or adjunctive therapy, is needed in the clinic considering its long duration and various observed adverse effects. Traditionally, *N. domestica*’s leaves, and fruits have long been used in the treatment of asthma, and chronic bronchitis in China. Remarkably, aqueous extract of leaves at the dose of 1 g/kg for 16 days showed beneficial effects on asthma symptoms, such as dyspnea, cough, and allergy in an ovalbumin (OVA)–specific asthma in guinea pig. Further investigation uncovered that alkaloids were the key constituents of the aqueous extract of leaves for asthma alleviation (Guo, et al., 2018). *N. domestica* additionally alleviated symptoms of asthma by relaxing smooth muscles. Tsukiyama et al. examined the impact of the aqueous extract from fruits and one of its constituents, nantenine (**23**), on histamine and serotonin-produced tracheal constriction in isolated guinea pigs. The findings demonstrated that histamine–induced competitive and non–competitive contractions were reduced by aqueous extract administration at doses ranging from 0.1 to 1 mg/mL. Moreover, aqueous extract treatment at the dose of 0.01–1 mg/mL suppressed serotonin–induced contractions in a competitive manner. Notably, nantenine (**23**) treatment at concentrations ranging from 2 to 20 μM did not impact histamine–induced contraction and only marginally decreased serotonin–induced contraction. The above–mentioned results showed that the aqueous extract had an inhibitory effect on tracheal smooth muscle contraction, whereas nantenine (**23**) alone could not explain the inhibitory effect of the aqueous extract on tracheal smooth muscle contraction (Tsukiyama, et al., 2007). Similarly, treatment with aqueous extract of fruits at a concentration of 1 mg/mL induced a biphasic relaxation of tracheal precontraction in response to high K^+^ stimulation in histamine–and serotonin–induced contraction of isolated guinea pig trachea. In actuality, the primary active components of the aqueous extract were higenamine (**50**) and nantenine (**23**). Furthermore, the aqueous extract of fruits relaxed tracheal smooth muscles immediately through *β*–adrenergic receptor stimulation by higenamine (**50**) and slowly through Ca^2+^ antagonism by nantenine (**23**) (Tsukiyama, et al., 2009; Ueki, et al., 2011). Taken together, the aqueous extract significantly inhibits the contraction of tracheal smooth muscle. However, further investigation is needed into the additional bioactive ingredients in aqueous extract besides nantenine (**23**).

### 5.8 Other effects

*N. domestica* possesses other effects in addition to the above-mentioned pharmacological activities. Specifically, a study found that nantenine (**23**), isolated from methanol extract of the fruits depressed serotonin–induced contraction of isolated rabbit aorta *via* suppression of serotonergic receptors (Shoji, et al., 1984). Another investigation showed that intraperitoneal injection of 13.3, 20, and 30 mg/kg nantenine (**23**) inhibited dose–dependently 5–hydroxy–L–tryptophan (l–5–HTP) plus clorgyline–induced head twitch response (HTR) in mice through blocking the central nervous system’s 5–HT2A receptors (Indra, et al., 2002). Notably, nantenine (**23**) suppressed adrenergic pressor responses in pithed rats through antagonizing *α*_1_–adrenergic, 5–HT2A and *α*_2_–adrenergic receptors in a concentration-dependent manner (Tsuchida and Ohizumi, 2003). Besides, the 95% ethanol extracts of *N. domestica*’s fruits and leaves exhibited insect repellent activity against *Aedes albopictus* with effective protection time of 2.94 h and 3.125 h, respectively. Therefore, the leaf and fruit extracts could be regarded as effective repellents against *A. albopictus* (Hu, et al., 2022).

## 6 Toxicity

*N*. *domestica* is a commonly grown decorative plant that is great for observing its leaves and fruits. Also, the aerial parts of *N*. *domestica* offers therapeutic benefits. However, the whole plant is also known for its toxic effects, especially the fruits which exhibit the highest level of toxicity. To date, two cyanohydrins, p–glucosyloxy–mandelonitrile (**362**) and nandinin (**363**) were found in young shoots and young leaves, respectively. The cyanohydrins are transformed into hydrogen cyanide when consumed, and this chemical paralyzes the nerve system. Even though *N. domestica* is believed to be toxic, little information about their possible toxicity is now accessible. Numerous cedar waxwing carcasses were found in Thomas County, Georgia, in April 2009, according to one case report. Out of them, a visual and microscopic examination of five cases revealed hemorrhage in the trachea, mediastinum, and lungs of the bird. Furthermore, the gastrointestinal tract contained the only food that had been consumed—wholly and partially digested *N. domestica* fruits. *N*. *domestica* fruits contained cyanide and were one of the few berries that could be the only food that was available at this time of year. The cedar waxwing was an omnivorous predator that often consumed more food than it could possibly need. The birds died from poisoning after consuming a hazardous amount of *N. domestica* fruits as a result of their voracious eating habits (Woldemeskel and Styer, 2010). According to another case, Texas, United States saw 875 incidents between 2000 and 2015 in which a patient, who was 5 years old or younger, consumed *N. domestica* fruits. There was a noticeable seasonal pattern in the amount of *N. domestica* fruits consumed, with March and April seeing the largest percentage of consumption. The most common clinical reactions were vomiting (3.7%), abdominal pain (1.0%), diarrhoea (0.9%), and nausea (0.7%) (Forrester, 2018). An *in vivo* toxicological experiment confirmed that the maximum daily dosage of the aqueous extract from *N. domestica* root was 122.4 g/kg, equivalent to 195.8 times the recommended clinical dosage. At this dosage, the mice showed no overt toxicological reactions and all of them lived for 14 days. Furthermore, during autopsy, no overt pathological alterations were found in the mice’s visceral organs. The aforementioned test results demonstrate that *N. domestica* root infusion administered by gavage has no discernible toxicological effects on mice, indicating that *N. domestica* root is a safe and dependable product (Qiu, et al., 2019). In general, people’s lives and health are closely related to the safety of pharmaceuticals. Therefore, to adequately expose the toxicity of the various medicinal parts of *N. domestica* and guarantee the safety of clinical use, more *in vivo* tests are required.

## 7 Conclusions and future perspectives

In the present review, we methodically compiled data on the traditional uses, phytochemistry, pharmacology, and toxicity of *N. domestica*. Traditionally, *N*. *domestica* have the effects of clearing away heat and dampness, energizing the meridians, relieving coughs and asthma. Through phytochemical research, at least 366 components have been found from *N*. *domestica* to date. Modern pharmacology demonstrates that *N*. *domestica* exhibits antitumor, dermatological, anti-inflammatory, antioxidant, antimicrobial, and detoxification activities, as well as effects on respiratory system, etc. These findings validate several traditional uses and clinical applications of TCM.

To further our understanding and utilization of *N. domestica*, we make the following suggestions. Firstly, nearly half of the components are detected from the volatile oil of *N*. *domestica* by GC-MS. The components in the volatile oil exist in the form of mixtures. There have been little investigations on the pharmacological properties of individual components in the volatile oil. Therefore, finding physiologically active natural compounds from the various medicinal portions of *N. domestica* by chromatographic techniques and *in vitro* and *in vivo* pharmacological activity investigations would be useful. Secondly, the existing pharmacological activity studies have focused on the crude extracts or semi-purified constituents of various medicinal parts of *N*. *domestica*. There should be a need for an in-depth exploration of the pharmacological activities of the chemical constituents isolated from *N*. *domestica*. Thirdly, *N*. *domestica* is traditionally used for the treatment of asthma, chronic bronchitis, conjunctivitis, whooping cough, pharyngeal tumors, etc. Existing pharmacological activity studies have demonstrated the anti-tumor, anti-inflammatory, antimicrobial, as well as effect on respiratory system, and so on. Numerous traditional uses of *N. domestica* have been validated by existing pharmacological activity. Therefore, further research is necessary to determine its precise mechanism. Fourthly, additional toxicity and pharmacokinetic investigations must be conducted on various medicinal parts and active ingredients isolated from *N*. *domestica* to validate their safety. Lastly, the quality markers of *N. domestica* should be determined based on the perspective of traditional application, pharmacological activity, toxicity, chemical composition, and blood components to identify a more rational and precise quality control system.

## Abbreviations

TCM, Traditional Chinese medicine; p53, Tumor antigen p53; PARP, Poly ADP–ribose polymerase; COX–2, Cyclooxygenase–2; p38, P38 mitogen-activated protein kinase; JNK, c-Jun N-terminal kinase; NF-κB, Nuclear factor-kappaB; ALT, Alanine aminotransferase; TNF–α, Tumor necrosis factor–alpha; CXCL–1, C–X–C motif chemokine 1; ADMA, Asymmetric dimethylarginine; IC_50_, Half maximal inhibitory concentration; CASP3/7, Caspase 3/7; LC3, Microtubule–associated protein 1 light chain 3; PGE2, Prostaglandin E2; MAPK, Mitogen–activated protein kinase; ERK, Extracellular signal-regulated kinase; AST, Aspartate aminotransferase; CFD, Coal, flay ash, and diesel exhausted particle; MIP–2, Macrophage inflammatory protein–2; SDMA, Symmetric dimethylarginine; l–5–HTP, 5–Hydroxy–L–tryptophan.
