# Supplementary material for: Nandina domestica Thunb.: a review of traditional uses, phytochemistry, pharmacology, and toxicology
Source: Front Pharmacol. 2024 Jul 9;15:1407140. doi: 10.3389/fphar.2024.1407140 (PMC11263726; doi:10.3389/fphar.2024.1407140)
Supplement: Supplementary file 1 [file Table1.pdf]

Table S1 Chemical compositions of *N. domestica*

No	Chemical composition	Plant part	Molecular formula	Reference
**Alkaloids**				
1	Berberine	Roots, aerial parts	$C_{20}H_{18}NO_4^+$	(Iwasa, et al. 2008; Tomita and Kitamura 1959)
2	Thalifendine	Stems, fruits	$C_{19}H_{16}NO_4^+$	(Ikuta and Itokawa 1988; Taha, et al. 2020)
3	Thalidastine	Stems	$C_{19}H_{16}NO_5^+$	(Ikuta and Itokawa 1988)
4	Berberastine	Stems	$C_{20}H_{18}NO_5^+$	(Ikuta and Itokawa 1988)
5	Coptisine	Stems, roots	$C_{19}H_{14}NO_4^+$	(Fu, et al. 2023; Ikuta and Itokawa 1988)
6	Epiberberine	Stems	$C_{20}H_{18}NO_4^+$	(Ikuta and Itokawa 1988)
7	Groenlandicne	Stems, fruits	$C_{19}H_{16}NO_4^+$	(Ikuta and Itokawa 1988; Taha, et al. 2020)
8	Palmatine	Stems, fruits	$C_{21}H_{22}NO_4^+$	(Ikuta and Itokawa 1988; Taha, et al. 2020)
9	Jatrorrhizine	Stems, fruits, roots	$C_{20}H_{20}NO_4^+$	(Ikuta and Itokawa 1988; Taha, et al. 2020;

				Tomita, et al. 1951)
10	Columbamine	Stems, fruits	$C_{20}H_{20}NO_4^+$	(Ikuta and Itokawa 1988; Taha, et al. 2020)
11	Dehydrodiscretamine	Stems	$C_{19}H_{18}NO_4^+$	(Ikuta and Itokawa 1988)
12	Demethyleneberberine	Fruits	$C_{19}H_{18}NO_4^+$	(Taha, et al. 2020)
13	Dehydrocorydaline	Fruits	$C_{22}H_{24}NO_4^+$	(Peng, et al. 2014b)
14	Pseudocolumbamine	Stems	$C_{20}H_{20}NO_4^+$	(Moriyasu, et al. 1992)
15	Desoxythalidastine	Stems	$C_{19}H_{14}NO_4^+$	(Ikuta and Itokawa 1988)
16	Stylopine	Fruits, roots	$C_{19}H_{17}O_4N$	(Cheng, et al. 2020; Fu, et al. 2023)
17	Sinactine	Fruits	$C_{20}H_{21}O_4N$	(Cheng, et al. 2020)
18	(*S*)–Scoulerine	Fruits	$C_{19}H_{21}NO_4$	(Taha, et al. 2020)
19	D–tetrahydropalmatine	Fruits	$C_{21}H_{25}NO_4$	(Cheng, et al. 2020)
20	Corydalmine	Fruits	$C_{20}H_{23}NO_4$	(Taha, et al. 2020)
21	Nandinine	Tree barks, fruits	$C_{19}H_{19}NO_4$	(Kunitomo, et al. 1974a; Taha, et al. 2020)
22	Tetrahydroberberine	Fruits	$C_{20}H_{21}NO_4$	(Taha, et al.

				2020)
23	*O*–methyldomesticine (Nantenine, Domestine)	Roots, aerial parts	$C_{20}H_{21}NO_4$	(Moriyasu, et al. 1992; Tomita and Kitamura 1959)
24	Nornantenine	Fruits, tree barks	$C_{19}H_{19}NO_4$	(Kunitomo and Murakami 1979; Kunitomo, et al. 1974b)
25	Hydroxynantenine	Roots, fruits	$C_{20}H_{21}NO_5$	(Cheng, et al. 2020; Fu, et al. 2023)
26	Domesticine	Roots, aerial parts	$C_{19}H_{19}NO_4$	(Iwasa, et al. 2008; Tomita and Kitamura 1959)
27	6R,6as–*N*–nantenine N_β–oxide	Seeds	$C_{20}H_{21}NO_5$	(Qin, et al. 2021)
28	6S,6as–*N*–nantenine N_α–oxide	Seeds	$C_{20}H_{21}NO_5$	(Qin, et al. 2021)
29	*O*–Methylbulbocapnine	Fruits	$C_{20}H_{21}NO_4$	(Peng, et al. 2014b)
30	Zizyphusine	Root barks	$C_{20}H_{24}NO_4^+$	(Funayama, et al. 1996)
31	Menisperine	Root barks, aerial parts	$C_{21}H_{26}NO_4^+$	(Funayama, et al. 1996; Iwasa, et al. 2008)
32	Magnoflorine	Stems, fruits	$C_{20}H_{24}NO_4^+$	(Kunitomo, et al. 1972; Tomita and Kugo 1956)
33	Glaucine	Fruits	$C_{21}H_{25}NO_4$	(Peng, et al. 2014b)
34	*N*–methyllaurotetanine	Seeds	$C_{20}H_{23}NO_4$	(Qin, et al. 2021)
35	Isoboldine	Aerial parts	$C_{19}H_{21}NO_4$	(Iwasa, et al.

				2008)
36	Isocorydine	Fruits	$C_{20}H_{23}NO_4$	(Kunitomo, et al. 1972)
37	Nuciferine	Roots	$C_{19}H_{21}NO_2$	(Fu, et al. 2023)
38	(+)–10–*O*–Methylhernovine N$_\beta$–oxide	Seeds	$C_{20}H_{23}NO_5$	(Qin, et al. 2021)
39	Oxoglaucidaline	Roots	$C_{21}H_{20}NO_6^+$	(Fu, et al. 2023)
40	Nandazurine	Roots, Stem bark	$C_{19}H_{14}NO_5^+$	(Tomita and Kitamura 1959)
41	Oxonantenine	Seeds, roots, fruits	$C_{19}H_{13}NO_5$	(Fu, et al. 2023; Kunitomo and Murakami 1979; Qin, et al. 2021)
42	Oxoglaucine	Roots	$C_{20}H_{17}NO_5$	(Fu, et al. 2023)
43	2–Hydroxy–1,9,10–trimethoxy–7H–dibenzo [de,g] quinoline–7–one	Roots	$C_{19}H_{15}NO_5$	(Fu, et al. 2023)
44	Didehydroglaucine	Fruits<br>Roots	$C_{21}H_{23}NO_4$	(Peng, et al. 2014b)<br>(Fu, et al. 2023)
45	Dehydronantenine	Tree barks, fruits, roots	$C_{20}H_{19}NO_4$	(Fu, et al. 2023; Kunitomo and Murakami 1979; Kunitomo, et al. 1975)
46	4,5–Dioxodehydronantenine	Fruits	$C_{20}H_{15}NO_6$	(Kunitomo and Murakami 1979)
47	*O*–Methylpallidine	Aerial parts	$C_{20}H_{23}NO_4$	(Iwasa, et al. 2008)
48	Sinoacutine	Aerial parts	$C_{19}H_{21}NO_4$	(Iwasa, et al. 2008)

49	*N*–methyl–2,3,6–trimethoxymorphinan–dien–7–one N_β–oxide	Seeds, roots	$C_{20}H_{23}NO_5$	(Fu, et al. 2023; Qin, et al. 2021)
50	Higenamine	Fruits	$C_{16}H_{17}NO_3$	(Cheng, et al. 2020)
51	(*R*)–Carnegine	Stems	$C_{13}H_{19}NO_2$	(Iwasa, et al. 2008)
52	Protopine	Fruits	$C_{20}H_{19}NO_5$	(Moriyasu, et al. 1992)
53	Allocryptopine	Fruits	$C_{21}H_{23}NO_5$	(Taha, et al. 2020)
54	Desoxyfructose–5–methoxytryptamine	Fruits	$C_{17}H_{24}N_2O_6$	(Cheng, et al. 2020)
55	Indolizino carbazole	Flowers	$C_{17}H_{12}N_2$	(Bajpai, et al. 2008; Bajpai, et al. 2009b)
56	Methyl 5–Hydroxy– 2– pyridinecarboxylate	Fruits	$C_7H_7O_3N$	(Peng, et al. 2020)
57	Gentianine	Fruits	$C_{10}H_9NO_2$	(Cheng, et al. 2020)
58	*N*–Methyl–3–phenyl–*N*–[2(*S*),3*R*,4–trihydroxy–butyl]–acrylamide	Fruits	$C_{14}H_{19}NO_4$	(Peng, et al. 2020)
59	*N*–Methyl–3–phenyl–*N*–[2(*R*),3*R*,4–trihydroxy–butyl]–acrylamide	Fruits	$C_{14}H_{19}NO_4$	(Peng, et al. 2020)
60	*N*–(4–aminobuty)–3–(4–hydroxy–3–methoxyphenyl)acrylamide	Fruits	$C_{14}H_{20}N_2O_3$	(Cheng, et al. 2020)
61	*N*–[1–hydroxy–2–p–tolyl–ethyl]acetamide	Fruits	$C_{11}H_{15}NO_2$	(Cheng, et al. 2020)
62	Terrestribisamide	Stems	$C_{24}H_{28}N_2O_6$	(Iwasa, et al. 2008)

63	*N*–(3',4'–dimethoxycinnamoyl)anthranilic acid	/	$C_{18}H_{17}NO_5$	(Katoh, et al. 1996)
64	Nandsterine	Fruits	$C_{29}H_{49}NO$	(Peng, et al. 2014b)
65	Methyl–*E*–mangolamide	Fruits	$C_{21}H_{26}N_2O_5$	(Imahori, et al. 2021)
66	Methyl–*Z*–mangolamide	Fruits	$C_{21}H_{26}N_2O_5$	(Imahori, et al. 2021)
**Flavonoids**				
67	Quercetin	Leaves	$C_{15}H_{10}O_7$	(Taha, et al. 2019a)
68	Quercetrin	Fruits	$C_{21}H_{20}O_{11}$	(Taha, et al. 2020)
69	Isoquercetin	Fruits	$C_{21}H_{20}O_{12}$	(Taha, et al. 2020)
70	Rutin	Leaves	$C_{27}H_{30}O_{16}$	(Taha, et al. 2019a)
71	Kaempferol 3–*O*–*β*–glucoside	Fruits	$C_{21}H_{20}O_{11}$	(Taha, et al. 2020)
72	Afzelin	Roots	$C_{21}H_{20}O_{10}$	(Fu, et al. 2023)
73	Cyanidin 3–*O*-xylosylglucoside	Fruits	$C_{26}H_{29}O_{15}^{+}$	(Ishikura 1971)
74	Pelargonidin 3–*O*–*β*–glucoside	Fruits	$C_{21}H_{21}O_{10}^{+}$	(Ishikura 1971)
75	Pelargonidin 3-*O*-xylosylglucoside	Fruits	$C_{26}H_{29}O_{14}^{+}$	(Ishikura 1971)
76	3,8'–Biapigenin	Fruits	$C_{30}H_{18}O_{10}$	(Taha, et al. 2020)
77	Cupressuflavone	Fruits	$C_{30}H_{18}O_{10}$	(Taha, et al. 2020)
78	Robustaflavone	Fruits	$C_{30}H_{18}O_{10}$	(Jo, et al. 2019)
79	Amentoftavone	Leaves	$C_{30}H_{18}O_{10}$	(Morita, et al.

		Fruits		1974) (Bajpai, et al. 2019)
**Lignans**				
80	Gentioluteol	Seeds	$C_{21}H_{26}O_8$	(Shu, et al. 2013)
81	Berchemol	Seeds	$C_{20}H_{24}O_7$	(Shu, et al. 2013)
82	Berchemol–4'–*O*–*β*–D–glucoside	Seeds	$C_{26}H_{34}O_{12}$	(Shu, et al. 2013)
83	(–)–Episyringaresinol	Tree barks	$C_{22}H_{26}O_8$	(Kunitomo, et al. 1975)
84	Syringaresinol	Seeds	$C_{22}H_{26}O_8$	(Shu, et al. 2013)
85	Pinoresinol	Seeds	$C_{20}H_{22}O_6$	(Shu, et al. 2013)
86	Medioresinol	Seeds	$C_{21}H_{24}O_7$	(Shu, et al. 2013)
87	1–Hydroxypinoresinol	Seeds	$C_{20}H_{22}O_7$	(Shu, et al. 2013)
**Terpenoids**				
88	Linalool	Whole plants	$C_{10}H_{18}O$	(Zhang 2009)
89	Hotrienol	Fruits, leaves, flowers	$C_{10}H_{16}O$	(Wang, et al. 2022; Zhang, et al. 2014)
90	(*Z*)–Tagetenone	Fruits	$C_{10}H_{14}O$	(Wang, et al. 2022)
91	Geraniol	Fruits	$C_{10}H_{18}O$	(Bi, et al. 2016)
92	2,6–Dimethyl–1,7–octadiene–3–ol	Flowers	$C_{10}H_{18}O$	(Bajpai, et al. 2008; Bajpai, et al. 2009b)
93	Cosmene	Fruits, leaves, flowers	$C_{10}H_{14}$	(Wang, et al. 2022; Zhang, et al. 2014)
94	Geranyl isovalerate	Fruits, leaves	$C_{15}H_{26}O_2$	(Wang, et al. 2022)

95	Geranylacetone	Fruits, leaves	$C_{13}H_{22}O$	(Wang, et al. 2022)
96	2–Isopropylidene–5–methylhex–4–enal	Fruits, leave	$C_{10}H_{16}O$	(Wang, et al. 2022)
97	*α*–Ionene	Fruits, leaves	$C_{13}H_{20}O$	(Bi, et al. 2016; Wang, et al. 2022)
98	*β*–Ionone	Fruits, leaves	$C_{13}H_{20}O$	(Wang, et al. 2022)
99	*α*–Cyclocitral	Leaves	$C_{10}H_{16}O$	(Wang, et al. 2022)
100	*β*–Cyclocitral	Leaves	$C_{10}H_{16}O$	(Wang, et al. 2022)
101	*β*–Damascenone	Fruits, leaves	$C_{13}H_{18}O$	(Bi, et al. 2016; Wang, et al. 2022)
102	4–(2,6,6–Trimethylcyclohexa–1,3–dienyl)but–3–en–2–one	Leaves	$C_{13}H_{18}O$	(Wang, et al. 2022)
103	4–(2,4,4–Trimethyl–cyclohexa–1,5–dienyl)–but–3–en–2–one	Fruits, leaves	$C_{13}H_{18}O$	(Wang, et al. 2022)
104	Safranal	Fruits, leaves	$C_{10}H_{14}O$	(Wang, et al. 2022)
105	1,5,8–*p*–Menthatriene	Flowers	$C_{10}H_{14}$	(Zhang, et al. 2014)
106	(+)–trans–*p*–Mentha–2,8–dien–1–ol	Fruits, leaves	$C_{10}H_{16}O$	(Wang, et al. 2022)
107	p–Menth–1–en–9–al	Fruits, leaves	$C_{10}H_{16}O$	(Wang, et al. 2022)
108	Dihydrocarvone	Fruits	$C_{10}H_{16}O$	(Wang, et al. 2022)

109	Nerol oxide	Flowers, fruits, leaves	$C_{10}H_{18}O_2$	(Bi, et al. 2016; Wang, et al. 2022; Zhang, et al. 2014)
110	2,6,6–Trimethyl–2–vinyltetrahydropyran	Fruits, leaves	$C_{10}H_{18}O$	(Wang, et al. 2022)
111	1,2,3,3,4–Pentamethyl cyclopentene	Flowers	$C_{10}H_{18}$	(Bajpai, et al. 2008; Bajpai, et al. 2009b)
112	*α*–Terpineol	Flowers, fruits, leaves	$C_{10}H_{18}O$	(Bi, et al. 2016; Wang, et al. 2022; Zhang, et al. 2014)
113	Lilac aldehyde A	Fruits	$C_{10}H_{16}O_2$	(Wang, et al. 2022)
114	trans–Furan linalool oxide	Whole plants	$C_{10}H_{18}O_2$	(Zhang 2009)
115	cis–Linalool oxide	Whole plants	$C_{10}H_{18}O_2$	(Zhang 2009)
116	cis–5–Isopropenyl–2–methyl–2–vinyltetrahydrofuran	Fruits, leaves	$C_{10}H_{16}O$	(Wang, et al. 2022)
117	Loliolide	Fruits	$C_{11}H_{16}O_3$	(Imahori, et al. 2021)
118	2,6,6–Trimethyl–10–methylene–1–oxacene –[4.5] –8–maltene	Whole plants	$C_{13}H_{20}O$	(Zhang 2009)
119	2,6,10,10–Tetramethyl–1–oxaspiro [4.5] decan–6–ol	Fruits, leaves	$C_{13}H_{24}O_2$	(Wang, et al. 2022)
120	(1*α*,3a*α*,7α,7a*β*)–Octahydro–1,4,4,7a–tetramethyl–1,3a–epoxy–3aH–inden–7–ol	Fruits, leaves	$C_{13}H_{22}O_2$	(Wang, et al. 2022)
121	*α*–Pinene oxide	Fruits, leaves	$C_{10}H_{16}O$	(Wang, et al. 2022)
122	Thujyl alcohol	Fruits, leaves	$C_{10}H_{18}O$	(Wang, et al.

				2022)
123	Bornylene	Flowers	$C_{10}H_{16}$	(Zhang, et al. 2014)
124	3,7,11–Trimethyldodeca–1,6,10–trien–3–ol	Fruits, leaves	$C_{15}H_{26}O$	(Wang, et al. 2022)
125	3,7,11–Trimethyl–1–dodecanol	Leaves	$C_{15}H_{32}O$	(Wang, et al. 2022)
126	6,10,14–Trimethylpentadecan–2–one	Fruits, leaves	$C_{18}H_{36}O$	(Wang, et al. 2022)
127	Shyobunone	Leaves	$C_{15}H_{24}O$	(Wang, et al. 2022)
128	Germacrene D	Fruits<br>Flowers	$C_{15}H_{24}$	(Wang, et al. 2022)<br>(Zhang, et al. 2014)
129	(+)–Dehydrovomifoliol	Fruits	$C_{13}H_{18}O_3$	(Imahori, et al. 2021)
130	(6*R*,9*R*)–9–Hydroxy–4–megastigmen–3–one	Fruits	$C_{13}H_{22}O_2$	(Imahori, et al. 2021)
131	(6*S*,9*S*)–Roseoside	Fruits	$C_{19}H_{30}O_8$	(Imahori, et al. 2021)
132	(6*S*,9*R*)–Roseoside	Fruits	$C_{19}H_{30}O_8$	(Imahori, et al. 2021)
133	Nandinamegastigmane I	Fruits	$C_{20}H_{32}O_8$	(Imahori, et al. 2021)
134	Nandinamegastigmane II	Fruits	$C_{20}H_{32}O_8$	(Imahori, et al. 2021)
135	Nandinamegastigmane III	Fruits	$C_{20}H_{32}O_8$	(Imahori, et al. 2021)

136	Nandinamegastigmane IV	Fruits	$C_{21}H_{32}O_8$	(Imahori, et al. 2021)
137	*α*–Muurolene	Fruits	$C_{15}H_{24}$	(Wang, et al. 2022)
138	*α*–Cadinol	Fruits	$C_{15}H_{26}O$	(Wang, et al. 2022)
139	Tau–Cadinol	Fruits, leaves	$C_{15}H_{26}O$	(Wang, et al. 2022)
140	Delta–Cadinol	Fruits	$C_{15}H_{26}O$	(Wang, et al. 2022)
141	(+)–delta–Cadinene	Fruits	$C_{15}H_{24}$	(Wang, et al. 2022)
142	*α*–Calacorene	Fruits	$C_{15}H_{20}$	(Wang, et al. 2022)
143	Intermedeol	Fruits	$C_{15}H_{26}O$	(Wang, et al. 2022)
144	1,2,3,5,6,7,8,8a–Octahydro–1–methyl–6–methylene–4–(1–methylethyl) –naphthalene	Flowers	$C_{15}H_{24}$	(Zhang, et al. 2014)
145	*γ*–Gurjunene	Flowers	$C_{15}H_{24}$	(Zhang, et al. 2014)
146	Cuparene	Flowers	$C_{15}H_{22}$	(Zhang, et al. 2014)
147	*α*–Cubebene	Flowers	$C_{15}H_{24}$	(Zhang, et al. 2014)
148	*β*–Cubebene	Flowers	$C_{15}H_{24}$	(Zhang, et al. 2014)
149	β–Copaene	Fruits	$C_{15}H_{24}$	(Wang, et al. 2022)
150	*α*–Copaene	Flowers	$C_{15}H_{24}$	(Zhang, et al.

				2014)
151	Espatulenol	Flowers	$C_{15}H_{24}O$	(Zhang, et al. 2014)
152	Viridiflorene	Fruits, leaves	$C_{15}H_{24}$	(Wang, et al. 2022)
153	(1*S*,3a*R*,4*S*,7*R*,8a*S*)– Decahydro–1,5,5,8a–tetramethyl–1,4–methanoazulen–7–ol	Fruits, leaves	$C_{15}H_{26}O$	(Wang, et al. 2022)
154	Cedrol	Fruits, leaves	$C_{15}H_{26}O$	(Wang, et al. 2022)
155	*α*–Cedrene	Leaves	$C_{15}H_{24}$	(Wang, et al. 2022)
156	Phytol	Whole plants	$C_{20}H_{40}O$	(Zhang 2009)
157	Neophytadiene	Leaves	$C_{20}H_{38}$	(Hu, et al. 2022)
158	24–Methylene–3–oxocycloartane 13–carboxylic acid	Fruits	$C_{31}H_{48}O_3$	(Kodai, et al. 2010)
**Phenolic acids and their derivatives**				
159	Syringic acid	Seeds	$C_9H_{10}O_5$	(Peng, et al. 2014c)
160	Gallic acid	Seeds	$C_7H_6O_5$	(Peng, et al. 2014c)
161	*p*–Hydroxybenzoic acid	Seeds	$C_7H_6O_3$	(Peng, et al. 2014c)
162	Protocatechuic acid	Roots, seeds	$C_7H_6O_4$	(Fu, et al. 2023; Peng, et al. 2014c)
163	Abiol	Flowers	$C_8H_8O_3$	(Bajpai, et al. 2008; Bajpai, et al. 2009b)
164	3–Hydroxy–4–methoxyphenylacetic acid	Roots	$C_9H_{10}O_4$	(Fu, et al. 2023)

165	Caffeic acid	Fruits, leaves, seeds	$C_9H_8O_4$	(Peng, et al. 2014c; Taha, et al. 2019b; Zhang, et al. 2014)
166	Chlorogenic acid	Fruits, leaves	$C_{16}H_{18}O_9$	(Taha, et al. 2020; Taha, et al. 2019b)
167	Feruloylquinic acid	Fruits	$C_{17}H_{20}O_9$	(Taha, et al. 2020)
168	Bergenin	Seeds	$C_{14}H_{16}O_9$	(Peng, et al. 2014c)
169	Ellagic acid	Seeds	$C_{14}H_6O_8$	(Peng, et al. 2014c)
170	3,3'–Di–*O*–methylellagic acid–4–*O*–*β*–D–glucoside	Seeds	$C_{22}H_{20}O_{13}$	(Peng, et al. 2014c)
171	4–*β*–D–glucopyranosyloxybenzoic acid	Leaves	$C_{13}H_{16}O_8$	(Masuda, et al. 2007)
172	Gastrodin–7–*O*–trans–caffeoyl ester	Fruits	$C_{22}H_{24}O_{10}$	(Kulkarni, et al. 2015)
173	Nandinaside A	Fruits	$C_{22}H_{22}O_{10}$	(Kulkarni, et al. 2015)
174	Nantenoside A	Leaves	$C_{22}H_{22}O_9$	(Morita, et al. 1974)
175	Nantenoside B (4–formylphenyl–4–*O*–[3–(3,4–dihydroxyphenyl)propenoyl]–*β*–D–glucopyranoside)	Leaves, fruits	$C_{22}H_{22}O_{10}$	(Kulkarni, et al. 2015; Morita, et al. 1974)
176	4–*O*–*β*–D–glucopyranosylbenzyl–(*E*)–3–(3,4–dihydroxyphenyl)acrylate	Fruits	$C_{22}H_{24}O_9$	(Imahori, et al. 2021)
177	4–*O*–*β*–D–glucopyranosylbenzyl–(*Z*)–3–(3,4–dihydroxyphenyl)acrylate	Fruits	$C_{22}H_{24}O_9$	(Imahori, et al. 2021)

178	Dihydrocaffeic acid methyl ester	Roots	$C_{10}H_{12}O_4$	(Fu, et al. 2023)
179	Ethyl gallate	Roots, seeds	$C_9H_{10}O_5$	(Fu, et al. 2023; Peng, et al. 2014c)
**Fatty acids**				
180	Myristic acid	Seeds, flowers	$C_{14}H_{28}O_2$	(Wang, et al. 2014; Zhang, et al. 2014)
181	Palmitic acid	Seeds, fruits, leaves	$C_{16}H_{32}O_2$	(Bi, et al. 2016; Hu, et al. 2022; Ohta and Miyazaki 1951)
182	Heptadecanoic acid	Seeds	$C_{17}H_{34}O_2$	(Wang, et al. 2014)
183	Stearic acid	Seeds	$C_{18}H_{36}O_2$	(Ohta and Miyazaki 1951) (Wang, et al. 2014)
184	Arachidic acid	Seeds	$C_{20}H_{40}O_2$	(Wang, et al. 2014)
185	Behenic acid	Seeds	$C_{22}H_{44}O_2$	(Wang, et al. 2014)
186	2–Decyltetradecanoic acid	Seeds	$C_{24}H_{48}O_2$	(Wang, et al. 2014)
187	9–Hexadecenoic acid	Seeds	$C_{16}H_{30}O_2$	(Wang, et al. 2014)
188	Oleic acid	Seeds, fruits, leaves	$C_{18}H_{34}O_2$	(Bi, et al. 2016; Ohta and Miyazaki 1951; Wang, et al.

				2022)
189	Linoleic acid	Seeds, fruits	$C_{18}H_{32}O_2$	(Bi, et al. 2016; Ohta and Miyazaki 1951)
190	11–Eicosenoic acid	Seeds	$C_{20}H_{38}O_2$	(Wang, et al. 2014)
191	11,13–Eicosadienoic acid	Seeds	$C_{20}H_{36}O_2$	(Wang, et al. 2014)
**Others**				
192	Dodecane	Whole plants	$C_{12}H_{26}$	(Zhang 2009)
193	Tetradecane	Whole plants	$C_{14}H_{30}$	(Zhang 2009)
194	Pentadecane	Leaves	$C_{15}H_{32}$	(Zhao, et al. 2010)
195	*N*–hexadecane	Whole plants	$C_{16}H_{34}$	(Zhang 2009)
196	*N*–heptadecane	Whole plants	$C_{17}H_{36}$	(Zhang 2009)
197	Octadecane	Whole plants	$C_{18}H_{38}$	(Zhang 2009)
198	Eicosane	Whole plants	$C_{20}H_{42}$	(Zhang 2009)
199	Heneicosane	Flowers	$C_{21}H_{44}$	(Zhang, et al. 2014)
200	Docosane	Fruits	$C_{22}H_{46}$	(Bi, et al. 2016)
201	Tricosane	Fruits	$C_{23}H_{48}$	(Bi, et al. 2016)
202	Tetracosane	Whole plants	$C_{24}H_{50}$	(Zhang 2009)
203	Pentacosane	Whole plants	$C_{25}H_{52}$	(Zhang 2009)
204	Hexacosane	Whole plants	$C_{26}H_{54}$	(Zhang 2009)
205	Cyclohexadecane	Flowers	$C_{16}H_{32}$	(Zhang, et al. 2014)
206	1,2–Propadiene	Flowers	C_3H_4	(Bajpai, et al. 2008; Bajpai, et al. 2009b)

207	Ethylidenecyclopropane	Flowers	C_5H_8	(Bajpai, et al. 2008; Bajpai, et al. 2009b)
208	1,3–Octadiene	Fruits, leaves	C_8H_{14}	(Wang, et al. 2022)
209	1,6–Dimethylhepta–1,3,5–triene	Fruits, leaves	C_9H_{14}	(Wang, et al. 2022)
210	*cis*,*cis*–1,3,5–Octatriene	Leaves	C_8H_{12}	(Wang, et al. 2022)
211	1–Hexadecene	Flowers	$C_{16}H_{32}$	(Zhang, et al. 2014)
212	1–Octadecene	Flowers	$C_{18}H_{36}$	(Zhang, et al. 2014)
213	(*Z*)–5–Nonadecene	Fruits	$C_{19}H_{38}$	(Bi, et al. 2016)
214	4–Nonyne	Flowers	C_9H_{16}	(Bajpai, et al. 2008; Bajpai, et al. 2009b)
215	Toluene	Flowers	C_7H_8	(Bajpai, et al. 2008; Bajpai, et al. 2009b)
216	Ethylbenzene	Fruits, leaves	C_8H_{10}	(Wang, et al. 2022)
217	(1–Methylethyl)–benzene	Fruits	C_9H_{12}	(Wang, et al. 2022)
218	Propyl–benzene	Flowers	C_9H_{12}	(Zhang, et al. 2014)
219	*O*–Xylene	Fruits, leaves	C_8H_{10}	(Wang, et al. 2022)
220	1,3–Dimethyl–benzene	Fruits, leaves	C_8H_{10}	(Wang, et al. 2022)

221	1–(But–3–en–2–yl)–4–methylbenzene	Leaves	$C_{11}H_{14}$	(Wang, et al. 2022)
222	1–(1–Methylethenyl)–2,3,4,5–tetramethylbenzene	Leaves	$C_{13}H_{18}$	(Wang, et al. 2022)
223	(*E*)–1–(2,3,6–Trimethylphenyl)buta–1,3–diene	Fruits, leaves	$C_{13}H_{16}$	(Wang, et al. 2022)
224	2–(2–Buten–1–yl)–1,3,5–trimethyl–benzene	Leaves	$C_{13}H_{18}$	(Wang, et al. 2022)
225	Naphthalene	Flowers	$C_{10}H_8$	(Bajpai, et al. 2008; Bajpai, et al. 2009b)
226	4–Isopropyl–1,6–dimethylnaphthalene	Fruits, leaves, flowers	$C_{15}H_{18}$	(Wang, et al. 2022; Zhang, et al. 2014)
227	1,1,6–Trimethyl–1,2–dihydronaphthalene	Fruits, leaves, flowers	$C_{13}H_{16}$	(Wang, et al. 2022; Zhang, et al. 2014)
228	1,2,3,4–Tetrahydro–1,1,6–trimethylnaphthalene	Leaves	$C_{13}H_{18}$	(Wang, et al. 2022)
229	1,2–Dihydro–2,5,8–trimethylnaphthalene	Fruits, leaves	$C_{13}H_{16}$	(Wang, et al. 2022)
230	Phenanthrene	Leaves	$C_{14}H_{10}$	(Wang, et al. 2022)
231	Methylcarbinol	Flowers	C_2H_6O	(Bajpai, et al. 2008; Bajpai, et al. 2009b)
232	Vinylcarbinol	Flowers	C_3H_6O	(Bajpai, et al. 2008; Bajpai, et al. 2009b)
233	3–Hexen–1–ol	Fruits	$C_6H_{12}O$	(Bi, et al. 2016)

234	Sorbic alcohol	Flowers	$C_6H_{10}O$	(Bajpai, et al. 2008; Bajpai, et al. 2009b)
235	Hexyl alcohol	Flowers	$C_6H_{14}O$	(Zhang, et al. 2014)
236	2–Nonyn–1–ol	Fruits, leaves	$C_9H_{16}O$	(Wang, et al. 2022)
237	*E*–2–Tetradecen–1–ol	Flowers	$C_{14}H_{28}O$	(Zhang, et al. 2014)
238	1–Dodecanol	Fruits	$C_{12}H_{26}O$	(Wang, et al. 2022)
239	2–Hexadecanol	Fruits, leaves	$C_{16}H_{34}O$	(Wang, et al. 2022)
240	2–Methyl–1–hexadecanol	Fruits, leaves	$C_{17}H_{36}O$	(Wang, et al. 2022)
241	2–[(Z)–9–Octadecenyloxy]ethanol	Leaves	$C_{20}H_{40}O_2$	(Wang, et al. 2022)
242	1–Heptatriacotanol	Fruits, leaves	$C_{37}H_{76}O$	(Wang, et al. 2022)
243	Brenzcatechin	Flowers	$C_6H_{12}O_2$	(Bajpai, et al. 2008; Bajpai, et al. 2009b)
244	(*Z*)–2–(3,3–Dimethylcyclohexylidene)ethanol	Fruits, leaves	$C_{10}H_{18}O$	(Wang, et al. 2022)
245	2–Furanmethanol	Whole plants	$C_5H_6O_2$	(Zhang 2009)
246	*α*–Toluenol	Flowers	C_7H_8O	(Bajpai, et al. 2008; Bajpai, et al. 2009b)
247	Phenylethyl alcohol	Whole plants	$C_8H_{10}O$	(Zhang 2009)

248	Mono phenol	Flowers	C_6H_6O	(Bajpai, et al. 2008; Bajpai, et al. 2009b)
249	Hydroquinone	Flowers	$C_6H_6O_2$	(Bajpai, et al. 2008; Bajpai, et al. 2009b)
250	*p*–Toluol	Flowers	C_7H_8O	(Bajpai, et al. 2008; Bajpai, et al. 2009b)
251	Dihydrochavicol	Flowers	$C_9H_{12}O$	(Bajpai, et al. 2008; Bajpai, et al. 2009b)
252	2–Methylphenol	Flowers	C_7H_8O	(Bajpai, et al. 2008; Bajpai, et al. 2009b)
253	Pyroguaiac acid	Flowers, fruits	$C_7H_8O_2$	(Bajpai, et al. 2008; Bajpai, et al. 2009b; Hu, et al. 2022)
254	Ortho–ethylphenol	Flowers	$C_8H_{10}O$	(Bajpai, et al. 2008; Bajpai, et al. 2009b)
255	4–Methyl catechol	Flowers	$C_7H_8O_2$	(Bajpai, et al. 2008; Bajpai, et al. 2009b)
256	2–Methoxy–4–vinylphenol	Fruits, flowers	$C_9H_{10}O_2$	(Bi, et al. 2016; Zhang, et al. 2014)
257	3,4–Xylenol	Flowers	$C_8H_{10}O$	(Bajpai, et al. 2008; Bajpai, et

				al. 2009b)
258	4–Ethylresorcinol	Flowers	$C_8H_{10}O_2$	(Bajpai, et al. 2008; Bajpai, et al. 2009b)
259	Orcinol	Flowers	$C_7H_8O_2$	(Bajpai, et al. 2008; Bajpai, et al. 2009b)
260	2,6–Di–*tert*–butyl–4–methylphenol	Whole plants	$C_{15}H_{24}O$	(Zhang 2009)
261	2,4–Di–*tert*–pentyphenol	Whole plants	$C_{16}H_{26}O$	(Zhang 2009)
262	Benzohydroquinone	Flowers	$C_{10}H_8O_2$	(Bajpai, et al. 2008; Bajpai, et al. 2009b)
263	4–Cumylphenol	Whole plants	$C_{15}H_{16}O$	(Zhang 2009)
264	1–Furyl–1–ethoxy–ethanol	Flowers	$C_8H_{12}O_3$	(Bajpai, et al. 2008; Bajpai, et al. 2009b)
265	Ethanone	Flowers	C_2H_4O	(Bajpai, et al. 2008; Bajpai, et al. 2009b)
266	2–Butanone	Flowers	C_4H_8O	(Bajpai, et al. 2008; Bajpai, et al. 2009b)
267	2–Pentanone	Flowers	$C_5H_{10}O$	(Bajpai, et al. 2008; Bajpai, et al. 2009b)
268	3–Pentanone	Flowers	$C_5H_{10}O$	(Bajpai, et al. 2008; Bajpai, et al. 2009b)
269	6–Methyl–5–hepten–2–one	Leaves	$C_8H_{14}O$	(Wang, et al. 2022)

270	3–Nonen–2–one	Fruits	$C_9H_{16}O$	(Wang, et al. 2022)
271	1–Decen–3–one	Leaves	$C_{10}H_{18}O$	(Zhao, et al. 2010)
272	2–Acetylfuran	Flowers	$C_6H_6O_2$	(Zhang, et al. 2014)
273	1–(1–Cyclohexen–1–yl)–ethanone	Leaves	$C_8H_{12}O$	(Zhao, et al. 2010)
274	Veltol	Flowers	$C_6H_6O_3$	(Bajpai, et al. 2008; Bajpai, et al. 2009b)
275	Ketoisophrone	Flowers	$C_9H_{12}O_2$	(Bajpai, et al. 2008; Bajpai, et al. 2009b)
276	2,2,6–Trimethylcyclohexanone	Leaves	$C_9H_{16}O$	(Wang, et al. 2022)
277	Suberon	Flowers	$C_7H_{12}O$	(Bajpai, et al. 2008; Bajpai, et al. 2009b)
278	Acetic acid	Flowers	$C_2H_4O_2$	(Bajpai, et al. 2008; Bajpai, et al. 2009b)
279	Glyoxylic acid	Flowers	$C_2H_2O_3$	(Bajpai, et al. 2008; Bajpai, et al. 2009b)
280	Isovaleric acid	Flowers, roots	$C_5H_{10}O_2$	(Bajpai, et al. 2008; Bajpai, et al. 2009b; Fu, et al. 2023)
281	Propionic acid	Flowers	$C_3H_6O_2$	(Bajpai, et al.

				2008; Bajpai, et al. 2009b)
282	Formic acid	Flowers	CH_2O_2	(Bajpai, et al. 2008; Bajpai, et al. 2009b)
283	Mandelic acid	Flowers	$C_8H_8O_3$	(Bajpai, et al. 2008; Bajpai, et al. 2009b)
284	Quinol dimethyl ether	Flowers	$C_8H_{10}O_2$	(Bajpai, et al. 2008; Bajpai, et al. 2009b)
285	*p*–Hydoxyanisole	Flowers	$C_7H_8O_2$	(Bajpai, et al. 2008; Bajpai, et al. 2009b)
286	Phenetole	Flowers	$C_8H_{10}O$	(Bajpai, et al. 2008; Bajpai, et al. 2009b)
287	2,4–Dimethylfuran	Flowers	C_6H_8O	(Bajpai, et al. 2008; Bajpai, et al. 2009b)
288	2–Pentylfuran	Fruits, leaves, flowers	$C_9H_{14}O$	(Wang, et al. 2022; Zhang, et al. 2014)
289	*trans*–2–(2–Pentenyl)furan	Fruits, leaves	$C_9H_{12}O$	(Wang, et al. 2022)
290	Tetrahydropyran	Flowers	$C_5H_{10}O$	(Bajpai, et al. 2008; Bajpai, et al. 2009b)
291	2,3–Dihydropyran	Flowers	C_5H_8O	(Bajpai, et al. 2008; Bajpai, et

				al. 2009b)
292	Benzofuran	Flowers	C_8H_6O	(Bajpai, et al. 2008; Bajpai, et al. 2009b)
293	2,3–Dihydrobenzofuran	Flowers	C_8H_8O	(Zhang, et al. 2014)
294	Safrole	Fruits	$C_{10}H_{10}O_2$	(Bi, et al. 2016)
295	2,7–Dimethyloxepine	Fruits, leaves	$C_8H_{10}O$	(Wang, et al. 2022)
296	Hexanal	Fruits, leaves	$C_6H_{12}O$	(Bi, et al. 2016; Wang, et al. 2022)
297	*trans*–2–Heptenal	Fruits, leaves	$C_6H_{10}O$	(Wang, et al. 2022)
298	Sorbaldehyde	Flowers	C_6H_8O	(Bajpai, et al. 2008; Bajpai, et al. 2009b)
299	*trans*–2,4–Heptadienal	Flowers, leaves	$C_7H_{10}O$	(Zhang, et al. 2014; Zhao, et al. 2010)
300	(*E*)–2–Octenal	Leaves	$C_8H_{14}O$	(Zhao, et al. 2010)
301	Nonanal	Fruits, leaves, flowers	$C_9H_{18}O$	(Wang, et al. 2022; Zhang, et al. 2014)
302	*trans*–2–Nonenal	Fruits, leaves	$C_9H_{16}O$	(Wang, et al. 2022)
303	Decanal	Fruits, leaves	$C_{10}H_{20}O$	(Wang, et al. 2022)
304	Hexadecanal	Flowers	$C_{16}H_{32}O$	(Zhang, et al.

				2014)
305	*E*–15–Heptadecenal	Flowers	$C_{16}H_{32}$	(Zhang, et al. 2014)
306	Octadecanal	Flowers	$C_{18}H_{36}O$	(Zhang, et al. 2014)
307	3–Furaldehyde	Flowers	$C_5H_4O_2$	(Zhang, et al. 2014)
308	Furfural	Flowers, fruits, leaves	$C_5H_4O_2$	(Bajpai, et al. 2008; Bajpai, et al. 2009b; Bi, et al. 2016; Hu, et al. 2022)
309	5–Methyl furfural	Flowers	$C_6H_6O_2$	(Zhang, et al. 2014)
310	Phenylacetaldehyde	Whole plants	C_8H_8O	(Zhang 2009)
311	*p*–Hydroxybenzaldehyde	Leaves	$C_7H_6O_2$	(Han, et al. 2011)
312	Benzaldehyde	Fruits	C_7H_6O	(Hu, et al. 2022)
313	Octyl formate	Flowers	$C_9H_{18}O_2$	(Zhang, et al. 2014)
314	Methyl palmitate	Flowers	$C_{17}H_{34}O_2$	(Zhang, et al. 2014)
315	Hexadecanoic acid, ethyl ester	Fruits, flowers	$C_{18}H_{36}O_2$	(Hu, et al. 2022; Zhang, et al. 2014)
316	Methyl stearate	Flowers	$C_{19}H_{38}O_2$	(Zhang, et al. 2014)
317	(*Z*,*Z*,*Z*)–9,12,15–Octadecatrienoic acid, methyl ester	Leaves, flowers	$C_{19}H_{32}O_2$	(Hu, et al. 2022; Zhang, et al. 2014)

318	(*E*,*E*)–9,12–Octadecadienoic acid, methyl ester	Leaves, flowers	$C_{19}H_{34}O_2$	(Hu, et al. 2022; Zhang, et al. 2014)
319	Ethyl–9,12,15–octadecatrienoate	Flowers	$C_{20}H_{34}O_2$	(Zhang, et al. 2014)
320	Propylene carbonate	Flowers	$C_4H_6O_3$	(Bajpai, et al. 2008; Bajpai, et al. 2009b)
321	Cyclohexanol, 3,3,5–trimethyl–, acetate, *cis*–	Leaves	$C_{11}H_{20}O_2$	(Zhao, et al. 2010)
322	Methyl benzoate	Roots, flowers	$C_8H_8O_2$	(Fu, et al. 2023; Zhang, et al. 2014)
323	5,5,8a–Trimethylhexahydro–2H–chromen–4a(5H)–yl acetate	Fruits, leaves	$C_{14}H_{24}O_3$	(Wang, et al. 2022)
324	(1*α*,2*β*,4*β*)–4–(1,1–Dimethylethyl)–1,2–cyclopentanedicarboxylic acid dimethyl ester	Fruits	$C_{13}H_{22}O_4$	(Wang, et al. 2022)
325	Methyl cinnamate	Flowers	$C_{10}H_{10}O_2$	(Zhang, et al. 2014)
326	Methyl salicylate	Flowers	$C_8H_8O_3$	(Zhang, et al. 2014)
327	Solbrol	Flowers	$C_8H_8O_3$	(Bajpai, et al. 2008; Bajpai, et al. 2009b)
328	Ethyl benzoate	Whole plants	$C_9H_{10}O_2$	(Zhang 2009)
329	Butyrolactone	Leaves	$C_4H_6O_2$	(Hu, et al. 2022)
330	Dibutyl phthalate	Roots	$C_{16}H_{22}O_4$	(Fu, et al. 2023)
331	Diisobutyl phthalate	Fruits, leaves	$C_{16}H_{22}O_4$	(Wang, et al. 2022)

332	2(3H)–Furanone	Flowers	$C_4H_4O_2$	(Bajpai, et al. 2008; Bajpai, et al. 2009b)
333	2(5H)–Furanone	Flowers	$C_4H_4O_2$	(Bajpai, et al. 2008; Bajpai, et al. 2009b)
334	Furandione	Flowers	$C_4H_2O_3$	(Bajpai, et al. 2008; Bajpai, et al. 2009b)
335	4–Oxy–5–methoxy–2–pentene–5–lactone	Whole plants	$C_6H_6O_4$	(Zhang 2009)
336	7,9–Di–*tert*–butyl–1–oxaspiro[4.5]deca–6,9–diene–2,8–dione	Whole plants	$C_{17}H_{24}O_3$	(Zhang 2009)
337	Dihydroactinidiolide	Whole plants	$C_{11}H_{16}O_2$	(Zhang 2009)
338	Glutaric anhydride	Whole plants	$C_5H_6O_3$	(Zhang 2009)
339	Aziridine	Flowers	C_2H_5N	(Bajpai, et al. 2008; Bajpai, et al. 2009b)
340	Leucine	Flowers	$C_6H_{13}NO_2$	(Bajpai, et al. 2008; Bajpai, et al. 2009b)
341	4–Pentenenitrile	Flowers	C_5H_7N	(Bajpai, et al. 2008; Bajpai, et al. 2009b)
342	*N*–Cyano–3–methylbut–2–enamine	Leaves	$C_6H_{10}N_2$	(Zhao, et al. 2010)
343	1–Nitrobutane	Flowers	$C_4H_9NO_2$	(Bajpai, et al. 2008; Bajpai, et al. 2009b)
344	Octyl azide	Flowers	$C_8H_{17}N_3$	(Bajpai, et al. 2008; Bajpai, et

				al. 2009b)
345	Erucylamide	Whole plants	$C_{22}H_{43}NO$	(Zhang 2009)
346	Pyrrolidine	Flowers	C_4H_9N	(Bajpai, et al. 2008; Bajpai, et al. 2009b)
347	2–Aminooxazole	Flowers	$C_3H_4N_2O$	(Bajpai, et al. 2008; Bajpai, et al. 2009b)
348	2–Ethylpyrrole	Flowers	C_6H_9N	(Zhang, et al. 2014)
349	2–Acetyl–1H–pyrrole	Whole plants	C_6H_7NO	(Zhang 2009)
350	3,5–Dimethylpyrazole	Flowers	$C_5H_8N_2$	(Bajpai, et al. 2008; Bajpai, et al. 2009b)
351	Pyrazole	Flowers	$C_3H_4N_2$	(Bajpai, et al. 2008; Bajpai, et al. 2009b)
352	Imidazole	Flowers	$C_3H_4N_2$	(Bajpai, et al. 2008; Bajpai, et al. 2009b)
353	3–Amino–1,2,4–triazole	Flowers	$C_2H_4N_4$	(Bajpai, et al. 2008; Bajpai, et al. 2009b)
354	1.2–D2–imidazole	Flowers	$C_3H_2D_2N_2$	(Bajpai, et al. 2008; Bajpai, et al. 2009b)
355	1–Methyltetrazole	Flowers	$C_2H_4N_4$	(Bajpai, et al. 2008; Bajpai, et al. 2009b)
356	Pyrazine	Flowers	$C_4H_4N_2$	(Bajpai, et al.

				2008; Bajpai, et al. 2009b)
357	Nicotinic acid	Flowers	$C_6H_5NO_2$	(Bajpai, et al. 2008; Bajpai, et al. 2009b)
358	Fourrine	Flowers	$C_6H_8N_2$	(Bajpai, et al. 2008; Bajpai, et al. 2009b)
359	Benzothiazole	Whole plants	C_7H_5NS	(Zhang 2009)
360	Indole	Flowers	C_8H_7N	(Zhang, et al. 2014)
361	Pyridine	Fruits	C_5H_5N	(Hu, et al. 2022)
362	*p*–Glucosyloxy–mandelonitrile	Young shoots	$C_{14}H_{17}NO_7$	(Abrol, et al. 1966)
363	Nandinin	Young leaves	$C_{23}H_{23}NO_{10}$	(Olechno, et al. 1984)
364	Propanoyl chloride	Flowers	C_3H_5ClO	(Bajpai, et al. 2008; Bajpai, et al. 2009b)
365	4–Chlorooctane	Flowers	$C_8H_{17}Cl$	(Bajpai, et al. 2008; Bajpai, et al. 2009b)
366	Diethoxydiphenylsilane	Fruits, leaves	$C_{16}H_{20}O_2Si$	(Wang, et al. 2022)
