# Supplementary material for: Nandina domestica Thunb.: a review of traditional uses, phytochemistry, pharmacology, and toxicology
Source: Front Pharmacol. 2024 Jul 9;15:1407140. doi: 10.3389/fphar.2024.1407140 (PMC11263726; doi:10.3389/fphar.2024.1407140)
Supplement: Supplementary file 2 [file Table2.pdf]

Table S2 Summary of the pharmacological activities of *N. domestica*

Test substance/part	Test system	Tested method	Positive drug	Results	References
**Antitumor**					
Chloroform and methanol extracts of root–barks, berberine	Human leukemia P388 cells (*in vitro*)	NA; NA	NA	Cytotoxicity with IC_{50} of 0.4, 1.5 and 3.0 μg/mL, respectively	(Funayama, et al. 1996)
Nandsterine	Human leukemia HL–60 cells (*in vitro*)	NA; 52 h	NA	Cytotoxicity with IC_{50} values of 52.1 μM	(Peng, et al. 2014b)
Oxonantenine, nantenine, nornantenine	Human non–small cell lung cancer A549 cells (*in vitro*)	NA; 48 h	Adriamycin	Oxonantenine was remarkably cytotoxic to A549 cells with an IC_{50} value of 8.15±0.34 μM Nantenine and nornantenine were moderately cytotoxic to A549 cells with IC_{50} values of 58.94±2.81 and 48.98±2.57 μM, respectively	(Qin, et al. 2021)
Methyl–*E*–mangolamide, methyl–*Z*–mangolamide	Human cervical carcinoma HeLa cells (*in vitro*)	60 μM; 3, 6, 12, 24 h	Adriamycin, Verapamil	Demonstrated cell–induced death activity on Adriamycin–treated HeLa cells	(Imahori, et al. 2021)
Protopine	Human colon cancer HCT116,	10, 20, 40 μM; 15, 48 h	NA	Increased p53–mediated transcriptional activity in a dose–dependent manner	(Son, et al. 2019)

	DLD–1, and HCT15 cells			Improved the phosphorylation of p53 at position Ser15, which helped to stabilize the p53 protein<br>Upregulated the expression of p53 downstream genes p21WAF1/CIP1 and BAX<br>Suppressed dose–dependently the proliferation of colon cancer HCT116 cells<br>Activated CASP3/7, catalyzed PARP cleavage and increased the number of Annexin V–FITC–positive cells<br>Stimulated autophagy in tumor cells via inducing LC3 puncta generation and LC3–II conversion	
**Dermatological activities**					
70% Ethanol extract of leaves	DNCB–induced AD NC/Nga mice model (*in vivo*)	300 mg/kg; 5 weeks	Dexamethasone	Significantly reduced skin thickness and mast cell infiltration in injured skin<br>Normalized the TCN in dorsal skin tissue, ALN, and spleen and decreased serum IgE levels and<br>Diminished the number of CD23+/B220+ cells and CD3+	(Yun, et al. 2021b)

				cells<br>Lowered the levels of IL–4/5/13, elevated the levels of interferon–γ in splenocytes, and upregulated notably claudin1 and Sirt1 protein expression	
Ethyl acetate fraction of the 70% ethanol extract from the leaves	DNCB induced eczema in mice (*in vivo*)	NA; 10d	Mometasone furoate	Exhibited anti–eczematic activity against DNCB induced eczema in mice<br>Superior to the positive control mometasone furoate in the treatment of eczema	(Taha, et al. 2019)
Volatile oil and the extracts (n–hexane, chloroform, ethyl acetate, and methanol) from flowers	*Trichophyton rubrum*, *Trichophyton mentagrophytes*, *Microsporum canis* (*in vitro*)	31.25, 62.5, 125, 250, 500, 1000 μg/mL; 30, 60, 90, 120, 150, 180 min	NA	Moderate to high antidermatophytic activity with inhibition of 51.90% ~ 68.60%<br>Essential oils exhibited inhibition of spore germination in all dermatophytes and inhibited *T. rubrum*	(Bajpai, et al. 2009a)
**Anti–inflammatory activity**					
Aqueous extract of fruits	LPS–stimulated human pulmonary epithelial A549 cells (*in*	1, 3, 10 μg/mL; 24 h	NA	Dose–dependently suppressed the expression of COX–2 and the production of PGE2 without affecting COX–1 expression and COX activity	(Ueki, et al. 2012)

	vitro)				
70% Ethanol extract of leaves	LPS–stimulated RAW 264.7 macrophages(*in vitro*)	10, 30, 100 μg/mL; 2, 12 h	Nordihydroguaiaretic acid	Decreased NO production and inhibited IL–6 and IL–1β mRNA expression<br>Suppressed phosphorylated activation of MAPK signaling pathways, encompassing ERK, p38 and JNK	(Yun, et al. 2020)
70% Ethanol extract of leaves	Fine dust CFD –induced lung injury mice model (*in vivo*)	100 mg/kg; 10 d	Dexa	Depressed IL–17, TNF–α, MIP–2 and CXCL–1<br>Reduced the accumulation of neutrophils<br>Facilitated histological alterations in lung tissue<br>Suppressed the elevation of ADMA and SDMA levels	(Yun, et al. 2021a)
Robustaflavone	LPS–stimulated RAW 264.7 macrophages (*in vivo*)	1, 2.5, 5, 10 μM; 24 h	NA	Decreased the production of NO, IL–1β and IL–6<br>Suppressed the expression of iNOS and COX–2, and down–regulated LPS–induced NF–κB expression and pERK 1/2	(Jo, et al. 2019)
Nandinaside A, nantenoside B	LPS–mediated inflammatory responses elicited in HUVEC cells	2.5, 5, 10, 20 μM; NA	NA	Suppressed LPS–induced leukocyte hyperpermeability, adhesion and migration on human endothelial cell monolayers	(Kulkarni, et al. 2015)

	(*in vivo*)				
**Antioxidant activity**					
80% Methanol extract of leaves	DPPH assay	0.25, 0.5, 1.0, 2.5 mg/mL; 20 min	Ascorbic acid	Showed DPPH radical scavenging capacity with IC_{50} of 0·403±0·013 mg/mL	(He, et al. 2003)
70% Ethanol extract of leaves	DPPH assay	10, 30, 100 μg/mL; 30 min	Ascorbic acid	DPPH radical scavenging activity in a dose–dependent manner	(Yun, et al. 2020)
Ethanol extract of leaves	Tyrosinase inhibitory activity assay	0.5, 0.15 mg/ml; 10 min	NA	Exhibited tyrosinase inhibitory activity with inhibitory rates of 81.5%±2.0% and 65.7±1.7%	(Masuda, et al. 2007)
Volatile oil from the flowers	ABTS, metal chelating ability assays	NA; 6, 10 min	Ascorbic acid, ethylene diamine tetraacetic acid	Produced scavenging effects on ABTS radicals as well as metal chelating ability	(Zhang, et al. 2014)
Volatile oil from fruits	DPPH, ABTS, and superoxide, reducing power, metal chelating ability assays	NA	Ascorbic acid	Exhibited reducing power with an IC_{50} value of 145.35±4.10 μg/mL<br>Displayed scavenging activity against DPPH and ATBS radicals with IC_{50} values of 28.39 ±1.12 and 20.61±0.75 μg/mL, respectively<br>Moderate scavenging activity against superoxide radicals with an IC_{50} value of 53.22 ±	(Bi, et al. 2016)

				2.51 μg/mL	
Amentoflavone	DPPH, ABTS, superoxide, and hydroxyl assays	10, 50, 100 μg/mL; NA	Quercetin	Scavenged DPPH, ABTS, superoxide, and hydroxyl radicals	(Bajpai, et al. 2019)
**Antimicrobial activity**					
95% Ethanol extract of leaves and stems	*S. aureus*, *S. faecalis*, *B. thuringiensis* (*in vitro*)	NA; 24 h	Gentamycin sulfate, nystatin	Exhibited antibacterial activity	(Li, et al. 2007; Li, et al. 2008)
Aqueous extract of leaves, ethanol, petroleum ether and ethyl acetate fractions	*S. aureus*, *E. coli*, *S. pyogenes*, *P. aeruginosa*, *A. baumannii* (*in vitro*)	1.5, 3, 6, 12, 24 mg/mL; 48 h	NA	Reduced the growth of both Gram–positive and Gram–negative bacteria by disrupting the bacterial membrane<br>Ethyl acetate fraction exhibited the best antibacterial activity<br>The alkaloids inhibited Gram–positive bacteria, whereas the flavonoids suppressed Gram–negative bacteria	(Guo, et al. 2018)
**Detoxification activity**					
Aqueous extract of seeds	Arsenic trioxide-induced hepatotoxicity and nephrotoxicit	20 g/kg; 14 d, 24 h	NA	Reduced structural damage to liver and renal tissue without hepatocellular necrosis<br>Lowered ALT, AST, Scr and BUN levels<br>Attenuated the decrease in	(Peng, et al. 2014a)

	y in rats (*in vivo*)			endogenous creatinine clearance rate<br>Decreased CAT levels and increased SOD and MDA levels in the homogenates of the renal cortex	
70% Ethanol extract of roots, stems, leaves, and fruits	arsenic trioxide induced hepatotoxicity and nephrotoxicity in rats (*in vivo*)	20 g/kg;10 d	NA	70% ethanol Extract of roots, stems, and fruits showed significant protective effects against arsenic trioxide - induced hepatotoxicity and nephrotoxicity, whereas *N. domestica's* leaves were slightly less effective	(Sun, et al. 2019)
Total alkaloids from the 70% ethanol extract of seeds	Arsenic trioxide induced acute liver injury in rats (*in vivo*)	0.7 g/kg; 7 d	NA	Significantly ameliorated arsenic trioxide-induced cardiac, renal, and hepatic damage	(Cheng, et al. 2020)
Different fractions (chloroform, ethyl acetate, n–butanol, and aqueous) from the 70% ethanol extract of roots	Arsenic trioxide induced acute liver and kidney injury in rats (*in vivo*)	20 g/kg; 10d	2,3–Dimercaptopropanesulfonic acid sodium salt	Protective effect against arsenic trioxide–induced hepatotoxicity and nephrotoxicity<br>Antagonistic effect on toxicity was observed with the chloroform fraction<br>Berberine showed a dose–dependent protective effect	(Fu, et al. 2023)

				against arsenic trioxide–induced hepatotoxicity and nephrotoxicity	
**Effect on respiratory system**					
Aqueous extract of leaves	OVA–stimulated asthma in guinea pig (*in vivo*)	1 g/kg/d; 16 d	Aminophylline	Showed beneficial effects on asthma symptoms, such as dyspnea, cough, and allergy	(Guo, et al. 2018)
Aqueous extract of fruits, higenamine, nantenine	Histamine– and serotonin–induced contraction of isolated guinea pig trachea (*in vivo*)	0.01, 0.1, 1 mg/mL, 2.16, 21.6, 216 nM, 0.2, 2, 20 μM; 60 min	Papaverine	Relaxed tracheal smooth muscles quickly through *β*–adrenergic receptor stimulation by higenamine and slowly Ca^{2+} antagonism by nantenine	(Tsukiyama, et al. 2009; Ueki, et al. 2011)
Aqueous extract of fruits, nantenine	Histamine– and serotonin–induced contraction in isolated guinea pig trachea (*in vitro*)	0.01, 0.1, 1 mg/mL, 0.2, 2, 20 μM; 10 min	Atropine, diphenhydramine	Inhibited histamine–induced competitive and non–competitive contractions<br>Suppressed serotonin–induced contractions in a competitive manner	(Tsukiyama, et al. 2007)
**Other effects**					

Nantenine	Isolated rabbit aorta (*in vivo*)	10, 30, 100 mM; NA	NA	Depressed serotonin–induced contraction of isolated rabbit aorta via suppression of serotonergic receptors	(Shoji, et al. 1984)
Nantenine	Male Wistar rats (*in vivo*)	0.03, 0.1, 0.3, 1, 3 mg/kg; 20, 40, 60, 80 min	NA	Suppressed adrenergic pressor responses in pithed rats through antagonizing α1–adrenergic, 5–HT2A and α_2–adrenergic receptors in a concentration-dependent manner	(Tsuchida and Ohizumi 2003)
Nantenine	l–5–HTP plus clorgyline–induced HTR in mice (*in vivo*)	13.3, 20, 30 mg/kg; 90 min	NA	Inhibited dose–dependently l–5–HTP plus clorgyline–induced HTR in mice through blocking the central nervous system's 5–HT2A receptors	(Indra, et al. 2002)
95% Ethanol extract of leaves and fruits	*Aedes albopictus* (*in vivo*)	NA	NA	Exhibited insect repellent activity against *Aedes albopictus* with effective protection time of 2.94 h and 3.125 h, respectively	(Hu, et al. 2022)

NA: Not Available; IC50: Half maximal inhibitory concentration; MIC: Minimum inhibitory concentration
